# Systematic understanding of anti-tumor mechanisms of Tamarixetin through network and experimental analyses

**DOI:** 10.1038/s41598-022-07087-6

**Published:** 2022-03-10

**Authors:** Sanu K. Shaji, G. Drishya, Damu Sunilkumar, Prashanth Suravajhala, Geetha B. Kumar, Bipin G. Nair

**Affiliations:** School of Biotechnology, Amrita Vishwa Vidyapeetham, Amritapuri, Clappana P.O, Kollam, Kerala 690525 India

**Keywords:** Cancer, Drug discovery, Systems biology

## Abstract

Tamarixetin, a flavonoid derived from Quercetin, was shown to possess anti-cancer properties in various types of cancer. However, the mechanism of action of this compound is not well understood. Observations from reverse docking and network pharmacology analysis, were validated by cell based studies to analyse the chemotherapeutic potential and elucidate the molecular mechanism of action of Tamarixetin in breast cancer. In silico analysis using reverse docking and PPI analysis clearly indicated that out of 35 proteins targeted by Tamarixetin, the top 3 hub genes, namely, AKT1, ESR1 and HSP90AA1, were upregulated in breast tumor tissues and more importantly showed strong negative correlation to breast cancer patient survival. Furthermore, the KEGG pathway analysis showed enrichment of target proteins of Tamarixetin in 33 pathways which are mainly involved in neoplastic signalling. In vitro cell-based studies demonstrated that Tamarixetin could inhibit cell proliferation, induce ROS and reduce mitochondrial membrane potential, leading to cell death. Tamarixetin induced cell cycle arrest at G2/M phase and inhibited the migration as well as the invasion of breast cancer cells. Taken together, the combination of in silico and in vitro approaches used in the present study clearly provides evidence for the chemotherapeutic potential of Tamarixetin in breast cancer.

## Introduction

Breast cancer is the most prominent type of cancer affecting the female population with an incident rate of 1 out of 10 women worldwide^1^. Both environmental as well as genetic factors contribute to the development of breast cancer. BRCA1 and BRCA2 mutations account for around 20% of familial breast cancer^2^. Germline mutations of p53 and ATM are also found to be responsible for breast cancer development^3^. PI3K/AKT/mTOR signal pathway activation confers breast tumor cells with proliferative advantages. It is estimated that approximately 70% of the tumors have hyper-activation of this pathway^4–6^. AKT1 activity is correlated with HER-2 overexpression in breast cancer which plays significant roles in mammary tumorigenesis by facilitating PI3K/AKT1 mediated transduction of HER-2 signalling cascades^7^. Around 70% of the breast cancer patients express estrogen and progesterone receptors^8^. Mutations in ESR1 gene that encodes for the estrogen receptor (ER) which are less observed in primary tumors (~ 1%) whereas such mutations are frequently observed (10–50%) in metastatic and endocrine therapy resistant tumors^9^.

Reverse docking is a sophisticated method to predict and analyse molecular targets of drugs and to help elucidate its mechanism of action. In reverse docking, a molecular virtual screen is performed to identify protein targets of a query ligand to predict the binding mode and binding affinity^10^. Reverse docking is a very promising strategy in drug discovery as it has wide applications such as identification of the targets of a compound, predicting the toxicity and adverse side effects of the drugs and repurposing of existing drugs^11^. Reverse docking combined with network pharmacology analysis can give insights into the drug–target network interactions^12^. Reverse docking is commonly used as an in silico modelling tool to elucidate anti-cancer mechanisms of many compounds such as Oxyresveratrol^13^, Cryptotanshinone (CT)^14^, 6-Methyl-1,3,8-trichlorodibenzofuran^15^, Epigallocatechin-3-Gallate^16^ and Quercetin^17^. After identification of the target genes using in silico methods, a network pharmacology approach was applied, to identify the comprehensive mechanism of action of the compound under investigation. Network pharmacology is an interdisciplinary discipline in the systematic research of drugs based on artificial intelligence and Big Data^18^. This approach uses principles of Systems Biology to identify the multiple pathways and biological processes that are affected by a compound. Hence, distant from “a single gene, single drug, single disease” conventional approach, network pharmacology focuses on poly-pharmacology with a “single drug, multiple genes, multiple effects” approach^19^. Studies using similar strategies were used to establish potential molecular mechanisms of active ingredients in various Traditional Chinese medicines (TCM). For example, network pharmacology analysis of nuciferine extracted from lotus leaves (used as raw material in traditional Chinese medicine) provided evidence for the antineoplastic mechanism of the compound against human neuroblastoma and mouse colorectal cancer^20^. Such studies propelled the translation of TCM from an experience based system to an evidence based system^21^.

Earlier studies from our laboratory had shown Tamarixetin, a naturally occurring flavonoid, to have anti-cancer as well as anti-metastatic properties in HT1080 cells^22^. Furthermore, Tamarixetin has also been shown to have antioxidant properties^23^. Our study demonstrated that the anti-metastatic potential of Tamarixetin is due to the downregulation of MMP-9 expression, mediated primarily through NFĸB inhibition. However, not much is known about the anti-cancer mechanism of this compound. Here, we employed reverse docking followed by a comprehensive network pharmacology analysis, supported by cell based studies for elucidation of the molecular mechanisms underlying the action of Tamarixetin in breast cancer.

The current study utilizes a multi-faceted approach of reverse docking, followed by network pharmacology approach, validated by cell based studies such as cell cycle analysis, cell proliferation, mitochondrial membrane potential perturbation, cell migration and invasion in 2D as well as 3D culture systems, for the identification and analyses of possible targets of Tamarixetin, in order to improve the understanding of the molecular mechanisms underlying the action of Tamarixetin in targeting breast cancer.

## Results

### Identification of candidate target genes of Tamarixetin in breast cancer

**Figure 1:**
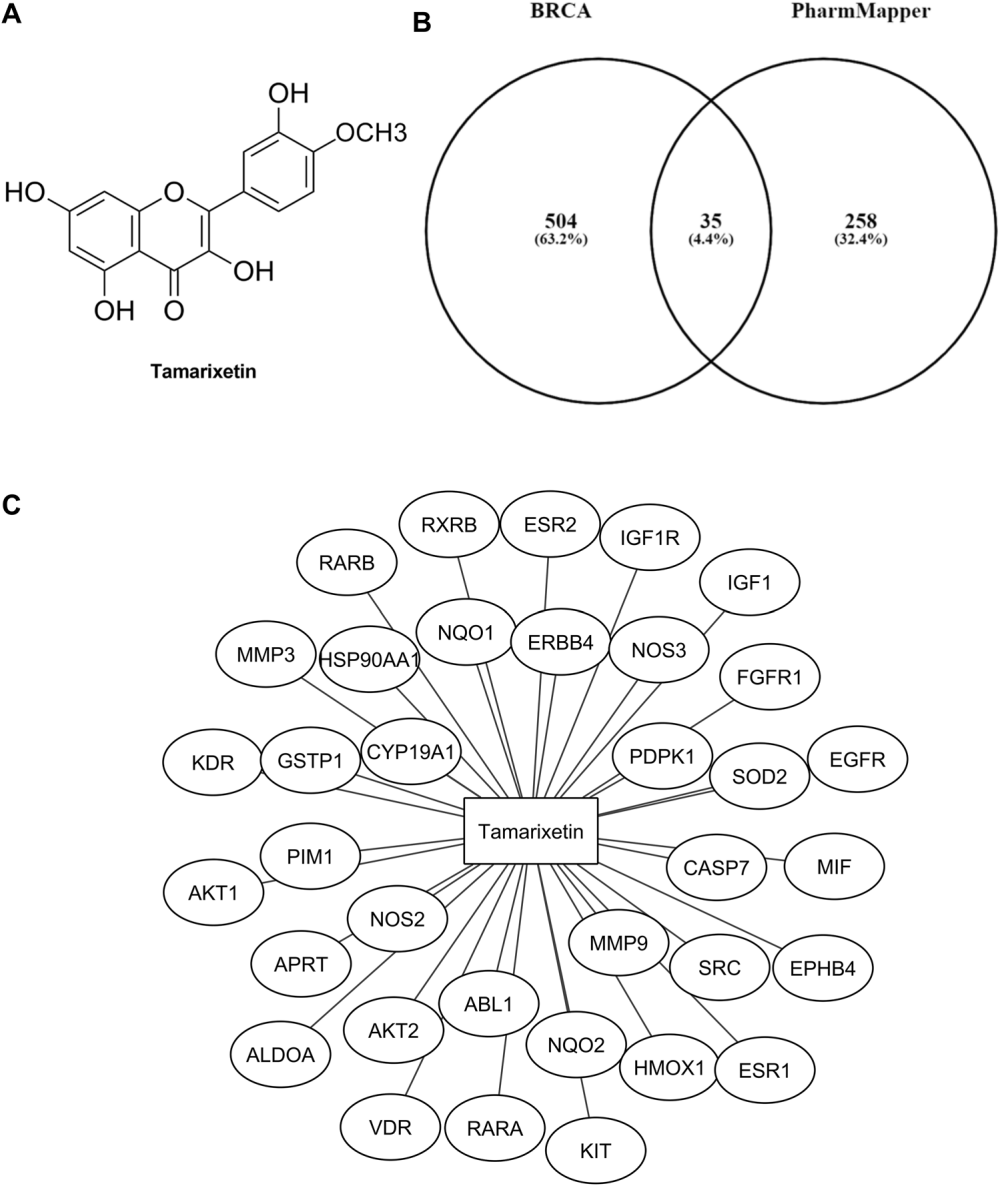
Identification of candidate target genes of Tamarixetin in breast cancer using reverse docking. (**A**) Structure of Tamarixetin. (**B**) Venn diagram representing breast cancer (BRCA) associated gees and Tamarixetin targets identified by reverse docking approach. (**C**) Target genes of Tamarixetin in breast cancer.

Reverse docking based target identification of human protein targets of Tamarixetin (Fig. 1A) was performed with the web-based tool PharmMapper. Around 293 predicted binding targets of Tamarixetin were obtained after removal of duplicate genes. Genes associated with breast cancer were obtained from DisGeNET, a curated database of disease-gene association. Gene disease association with strong evidence has a GDA score of ≥ 0.3^24^. Based on the cut off criteria, 539 breast cancer-associated genes were identified, in which 35 genes were found to be the target of Tamarixetin based on reverse docking analysis (Fig. 1B,C, Table 1). These 35 genes were further explored using a network pharmacology approach.

**Table 1:** Target genes of Tamarixetin in breast cancer.

Sl no	Protein name	DisGeNET GDA score	PharmMapper fit score
1	AKT1	0.7	2.544
2	ESR1	0.7	2.957
3	AKT2	0.6	2.62
4	FGFR1	0.6	5.996
5	PDPK1	0.55	3.55
6	ERBB4	0.5	4.05
7	NQO2	0.43	2.952
8	CYP19A1	0.4	2.975
9	NQO1	0.4	3.507
10	EGFR	0.4	2.796
11	EPHB4	0.4	3.975
12	ESR2	0.4	3.97
13	ABL1	0.4	4.79
14	GSTP1	0.4	2.585
15	HMOX1	0.4	3.545
16	HSP90AA1	0.4	3.979
17	IGF1	0.4	3.904
18	IGF1R	0.4	3.696
19	APRT	0.4	3.369
20	KDR	0.4	3.575
21	KIT	0.4	4.797
22	MMP3	0.4	2.904
23	MMP9	0.4	2.849
24	NOS3	0.4	3.042
25	RARA	0.4	3.009
26	RARB	0.4	3.327
27	SOD2	0.4	3.853
28	SRC	0.4	4.698
29	VDR	0.4	3.913
30	NOS2	0.39	2.866
31	MIF	0.38	3.31
32	PIM1	0.36	2.938
33	RXRB	0.31	4.519
34	CASP7	0.31	3.581
35	ALDOA	0.3	2.907

### Gene ontology analysis of Tamarixetin target genes in BRCA

In order to get a better understanding of the functional role of target genes, GO analysis was performed using DAVID software. Under Biological Process (BP) category (Supplementary Table S1), 68 GO terms were enriched within FDR cut off. More than 10 target genes were enriched in “GO:0046777 ~ protein autophosphorylation”, “GO:0043066 ~ negative regulation of apoptotic process” and “GO:0007165 ~ signal transduction” ontology. Most significantly enriched term in the BP category was “GO:0046777 ~ protein autophosphorylation”. In the Molecular Function (MF) category (Supplementary Table S2), 22 terms were enriched within FDR cut off. Majority of the proteins (31 target proteins) were enriched in “GO:0005515 ~ protein binding”, followed by “GO:0005524 ~ ATP binding” (14 target proteins) and “GO:0042802 ~ identical protein binding” (10 target genes). The most significantly enriched term in MF category was “GO:0004713 ~ protein tyrosine kinase activity”. In the Cellular Compartment (CC) category (Supplementary Table S3), 22 proteins were enriched in “GO:0005634 ~ nucleus”. followed by “GO:0005886 ~ plasma membrane” (18 proteins) and “GO:0005829 ~ cytosol” (17 proteins). Gene ontology analysis revealed that under BP as well as MF, majority of the terms enriched were associated with events connected to cancer progression.

### KEGG pathway analysis of Tamarixetin target genes

In order to identify significant pathways associated with Tamarixetin target genes, gene set enrichment analysis in the KEGG pathway database was performed (Table 2, Supplementary Table S4). The most significantly enriched pathway was “hsa05200:Pathways in cancer” with 15 target proteins (HSP90AA1, NOS2, GSTP1, IGF1, MMP9, EGFR, IGF1R, RXRB, AKT2, KIT, ABL1, RARA, AKT1, RARB, FGFR1), followed by “hsa05205:Proteoglycans in cancer” with 12 target genes (ERBB4, PDPK1, SRC, AKT2, KDR, AKT1, IGF1, ESR1, MMP9, EGFR, FGFR1, IGF1R). Of the total target genes, 11 were enriched in “hsa04151:PI3K-Akt signaling pathway” (HSP90AA1, PDPK1, NOS3, AKT2, KIT, KDR, AKT1, IGF1, EGFR, FGFR1, IGF1R).

**Table 2:** The top 10 significantly enriched pathways in the KEGG database.

Term	Count	FDR	Genes
hsa05200:Pathways in cancer	15	4.96E−08	HSP90AA1, NOS2, GSTP1, IGF1, MMP9, EGFR, IGF1R, RXRB, AKT2, KIT, ABL1, RARA, AKT1, RARB, FGFR1
hsa05205:Proteoglycans in cancer	12	4.96E−08	ERBB4, PDPK1, SRC, AKT2, KDR, AKT1, IGF1, ESR1, MMP9, EGFR, FGFR1, IGF1R
hsa04915:Estrogen signaling pathway	9	4.47E−07	HSP90AA1, SRC, NOS3, AKT2, AKT1, ESR1, MMP9, EGFR, ESR2
hsa05215:Prostate cancer	8	3.38E−06	HSP90AA1, PDPK1, AKT2, AKT1, IGF1, EGFR, FGFR1, IGF1R
hsa04066:HIF-1 signaling pathway	8	4.94E−06	NOS2, NOS3, AKT2, HMOX1, AKT1, IGF1, EGFR, IGF1R
hsa04151:PI3K-Akt signaling pathway	11	4.55E−05	HSP90AA1, PDPK1, NOS3, AKT2, KIT, KDR, AKT1, IGF1, EGFR, FGFR1, IGF1R
hsa04015:Rap1 signaling pathway	9	6.38E−05	SRC, AKT2, KIT, KDR, AKT1, IGF1, EGFR, FGFR1, IGF1R
hsa05223:Non-small cell lung cancer	6	6.99E−05	RXRB, PDPK1, AKT2, RARB, AKT1, EGFR
hsa04014:Ras signaling pathway	9	8.55E−05	AKT2, KIT, ABL1, KDR, AKT1, IGF1, EGFR, FGFR1, IGF1R
hsa05218:Melanoma	6	1.81E−04	AKT2, AKT1, IGF1, EGFR, FGFR1, IGF1R

### Protein–protein interaction network of Tamarixetin target genes

**Figure 2:**
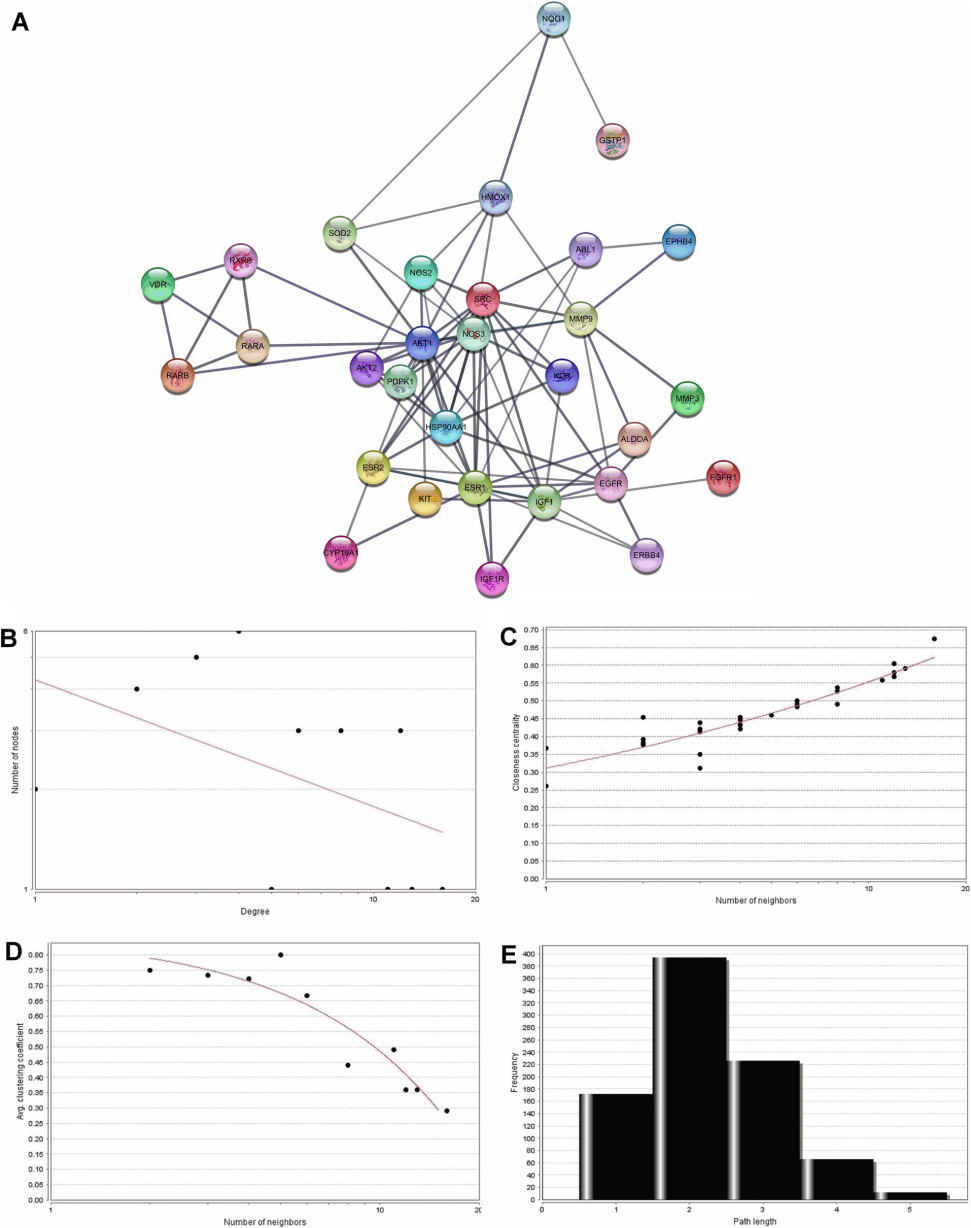
Protein–protein interaction network of Tamarixetin targets in breast cancer and its topological analysis. (**A**) PPI network of target genes constructed using the STRING database. (**B**) Distribution of degree. (**C**) Proximity to center. (**D**) Average aggregation coefficient. (**E**) Distribution of the shortest path.

The STRING database was used for the construction of protein–protein interaction (PPI) network of target genes. The resulting network showed a highly significant interaction among the proteins (p value < 1.0e^−16^). A significant interaction in the PPI network denotes that the proteins are at least partially connected, which increases the possibility of being regulated by similar mechanisms. The number of nodes in the PPI network was 35 with 86 edges, with an average node degree of 4.91 and average local clustering coefficient 0.553 (Fig. 2A). The network was found to have a diameter equal to 5 and radius equal to 3. Network centralization was 0.346 with a characteristic path length of 2.255, network density of 0.145 and network heterogeneity of 0.854. NetworkAnalyzer was used to identify node degree distribution (Fig. 2B), proximity to the center (Fig. 2C), aggregation coefficient (Fig. 2D) and shortest path length (Fig. 2E). The analysis showed that the PPI network of Tamarixetin target genes followed the characteristic of a small-world network, which is one of the main properties of the biological network. The network obtained was scale-free, obeying power-law distribution.

### Hub genes AKT1, ESR1 and HSP90AA1 are upregulated in breast cancer which is associated with reduced survival in breast cancer patients

**Figure 3:**
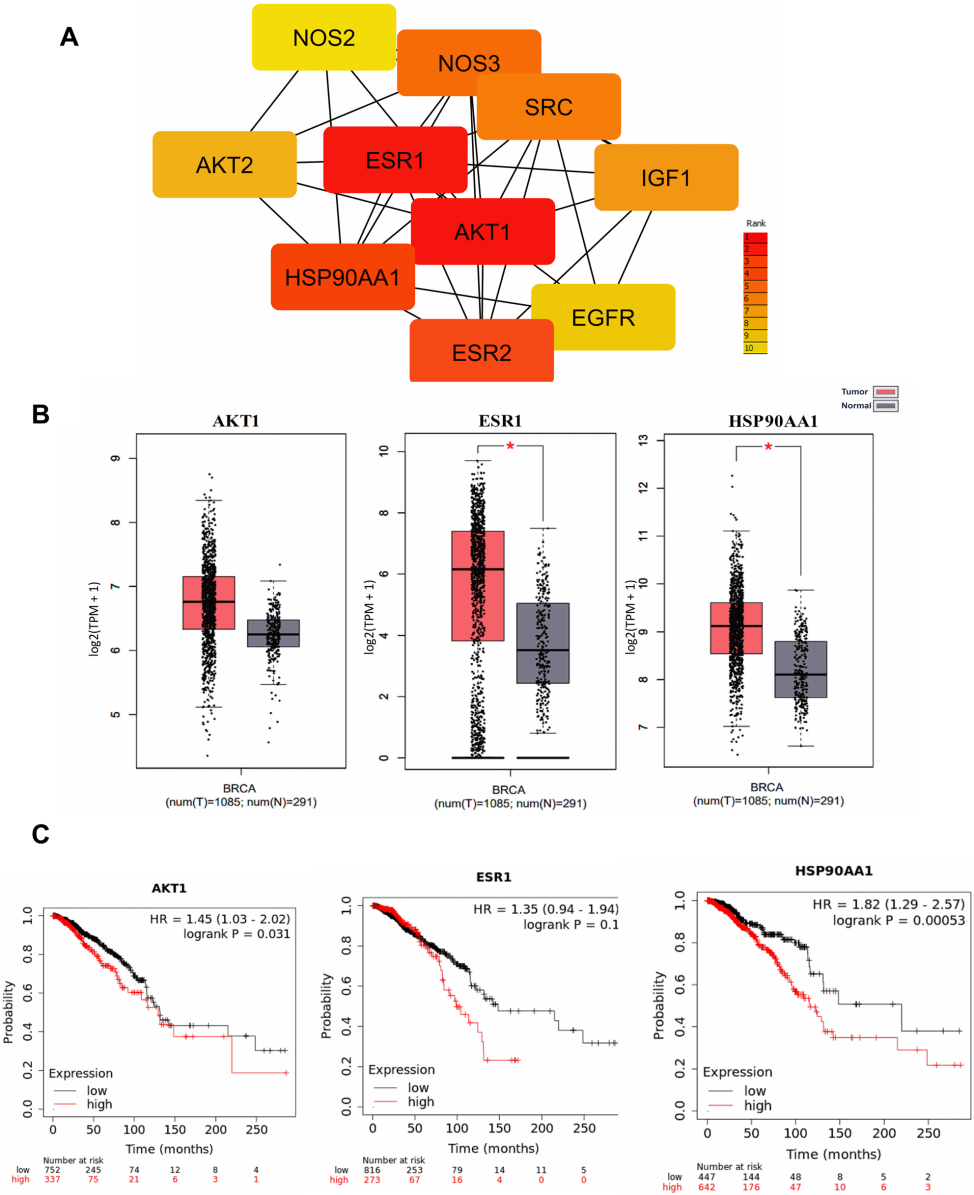
Hub genes in the PPI network. (**A**) Hub genes were identified using the cytoHubba plugin in the Cytoscape platform. (**B**) Expression profiles of AKT1, ESR1 and HSP90AA1. TCGA data were accessed through Gepia server for the analysis. (**C**) Survival analysis of top 3 hub genes in the PPI network. TCGA data were accessed through KMplotter pan-cancer analysis for the estimation of overall survival.

Cytoscape plugin cytoHubba was used to identify hub genes in the network. Hub genes are the nodes in the network with a high degree of connectivity. AKT1 was found to be the top-ranked hub gene followed by ESR1 and HSP90AA1 in the second and third position respectively (Fig. 3A). A detailed investigation on these top-ranked hub genes indicated its prominent role in tumor progression.

Gene expression data of breast cancer patients was accessed from TCGA using Gepia server. Expression data of normal breast tissues was obtained from both TCGA as well as GTEx databases. Analysis of the top 3 hub genes in the PPI network showed that all these genes are found to be upregulated in breast cancer tissues compared to normal breast tissue (Fig. 3B). The median expression of ESR1 and HSP90AA1 was significantly increased in tumor cells.

The analysis showed that high expression of all three genes were associated with poor survival in breast cancer patients (Fig. 3C). AKT1 showed a statistically significant (logrank P = 0.031) hazard ratio (HR) of 1.45 in BRCA patients with a median survival of 131.5 months in the low expression cohort versus 129.1 months in the high expression cohort. HSP90AA1 also showed statistically significant (logrank P = 0.00053) reduction in patient survival with increased expression and a hazard ratio of 1.82. The median survival of the low expression cohort was 219.77 months, whereas the high expression cohort showed a median survival of 115.73 months. Even though the Kaplan–Meier survival analysis of ESR1 in breast cancer showed a reduction in patient survival with increased expression, the result was not statistically significant (logrank P = 0.1). The hazard ratio of ESR1 was found to be 1.35, with a median survival of 148.53 months in the low expression cohort against 98.83 months in the high expression cohort.

### Two functional modules are identified in the PPI network

**Figure 4:**
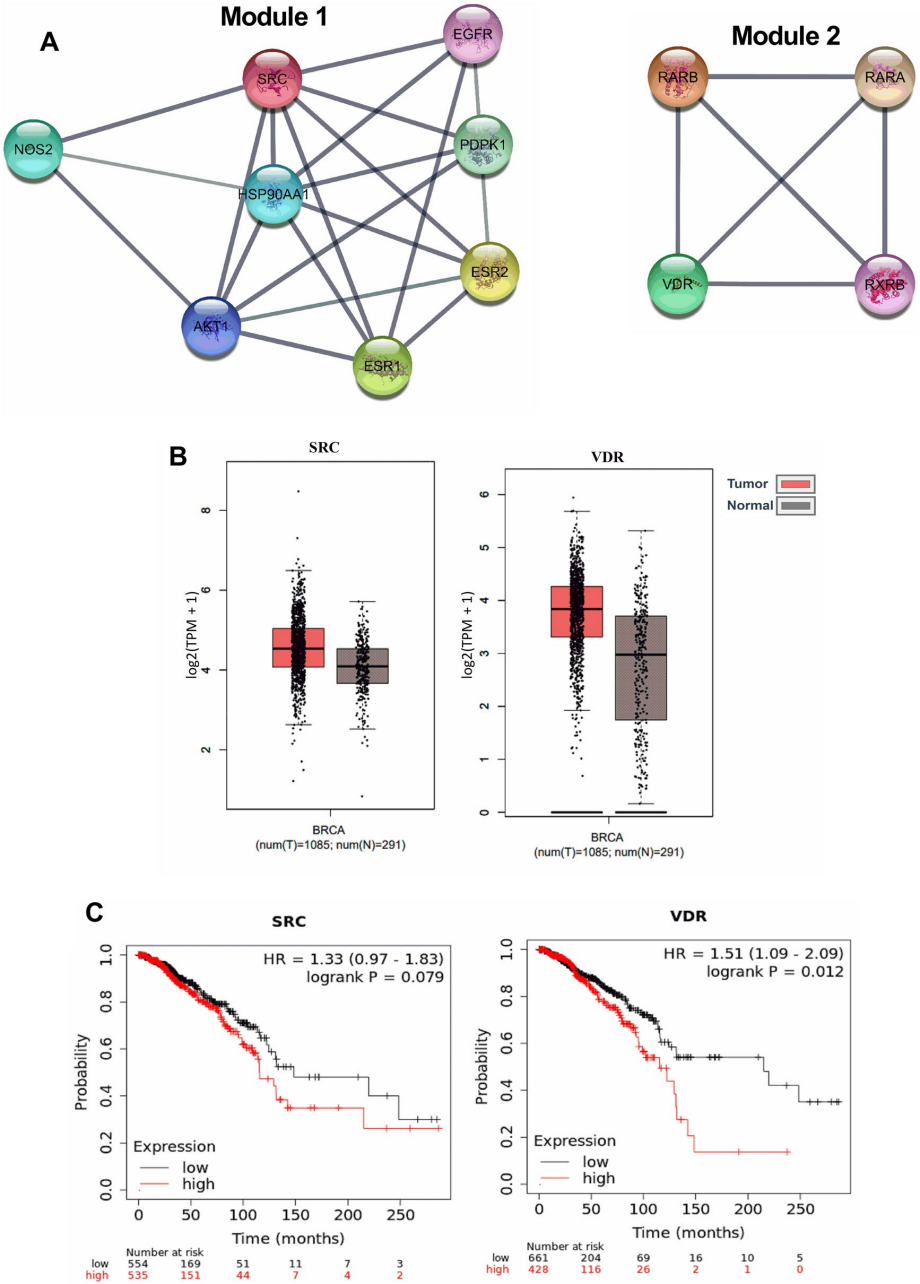
Functional modules in the PPI network. (**A**) MCODE analysis identified two functional modules in the network. (**B**) Expression analysis of SRC and VRD in normal and tumor breast tissues accessed from the TCGA database. (**C**) Survival analysis of SRC and VRD breast cancer patients.

Analysis of PPI data using MCODE algorithm in the Cytoscape platform identified two functional modules in the network. Module1 (Fig. 4A) with a cluster score of 5.714, possessed 8 nodes with 20 edges. It contained NOS2, EGFR, PDPK1, SRC, AKT1, ESR2, HSP90AA1 and ESR1. SRC was found to be the seed gene in this functional module with an MCODE score of 4.52. Functional module 2 consisted of 4 nodes (VDR, RXRB, RARA and RARB) and 6 edges with a cluster score of 4. The seed gene in the cluster was identified to be VDR with an MCODE score of 3. The seed genes in the functional modules, SRC and VDR were found to be upregulated in cancer tissue samples compared to normal breast samples (Fig. 4B). Even though a higher expression of SRC levels was found to be correlated with poor patient survival (hazard ratio 1.33), the log rank P value obtained was 0.079 indicating the lack of statistical significance in the observation (Fig. 4C). The median survival of the low expression cohort was also higher (148.53 months), compared to that of the high expression cohort (115.73 months). However, VDR showed a very significant association with patient survival. The hazard ratio was found to be 1.51 with a log rank P value of 0.012. The median survival of the BRCA patient with low expression of VDR was 215.2 months whereas the patients expressing higher levels of VDR had a median survival of only 115.37 months.

### Association of Tamarixetin target to cancer hallmarks

**Figure 5:**
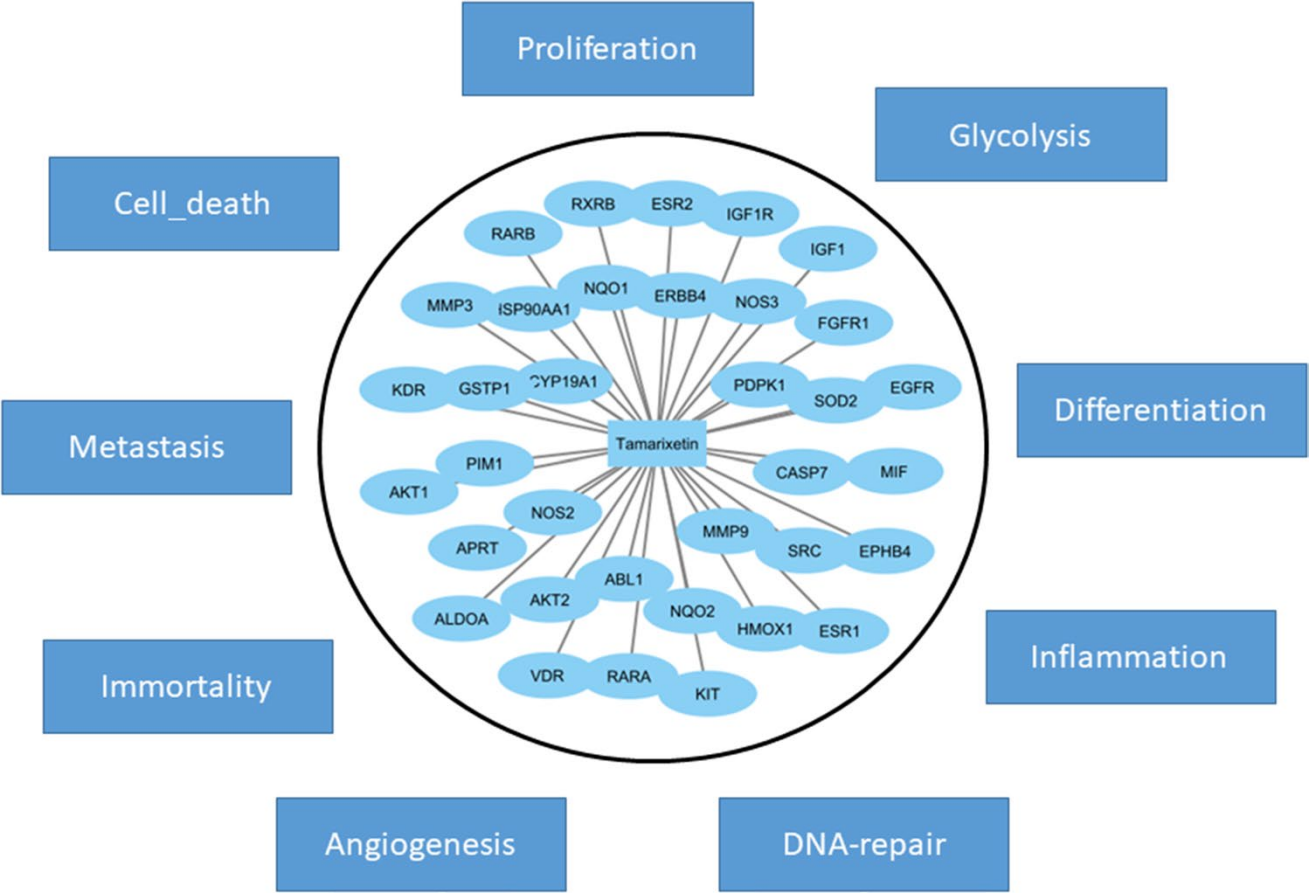
Association of Tamarixetin target genes to various hallmarks of cancer. The analysis was performed using CancerGeneNet server for the identification of association between cancer hallmarks and Tamarixetin target genes in breast cancer obtained by reverse docking.

In order to get a comprehensive understanding of the effect of Tamarixetin in various hallmarks of cancer, CancerGeneNet server was used. This server links cancer-associated genes to cancer hallmark phenotype^25^. Tamarixetin target genes were found to be associated with various cancer hallmarks such as Proliferation, Glycolysis, Differentiation, Inflammation, DNA-Repair, Angiogenesis, Immortality, Metastasis and Cell death (Fig. 5, Supplementary Fig. S1). These observations indicate that Tamarixetin could function as an anti-cancer agent by acting on multiple hallmarks of cancer and modulating its expression at multiple levels leading to the attenuation of cancer progression.

### Tamarixetin inhibits proliferation of breast cancer cells

**Figure 6:**
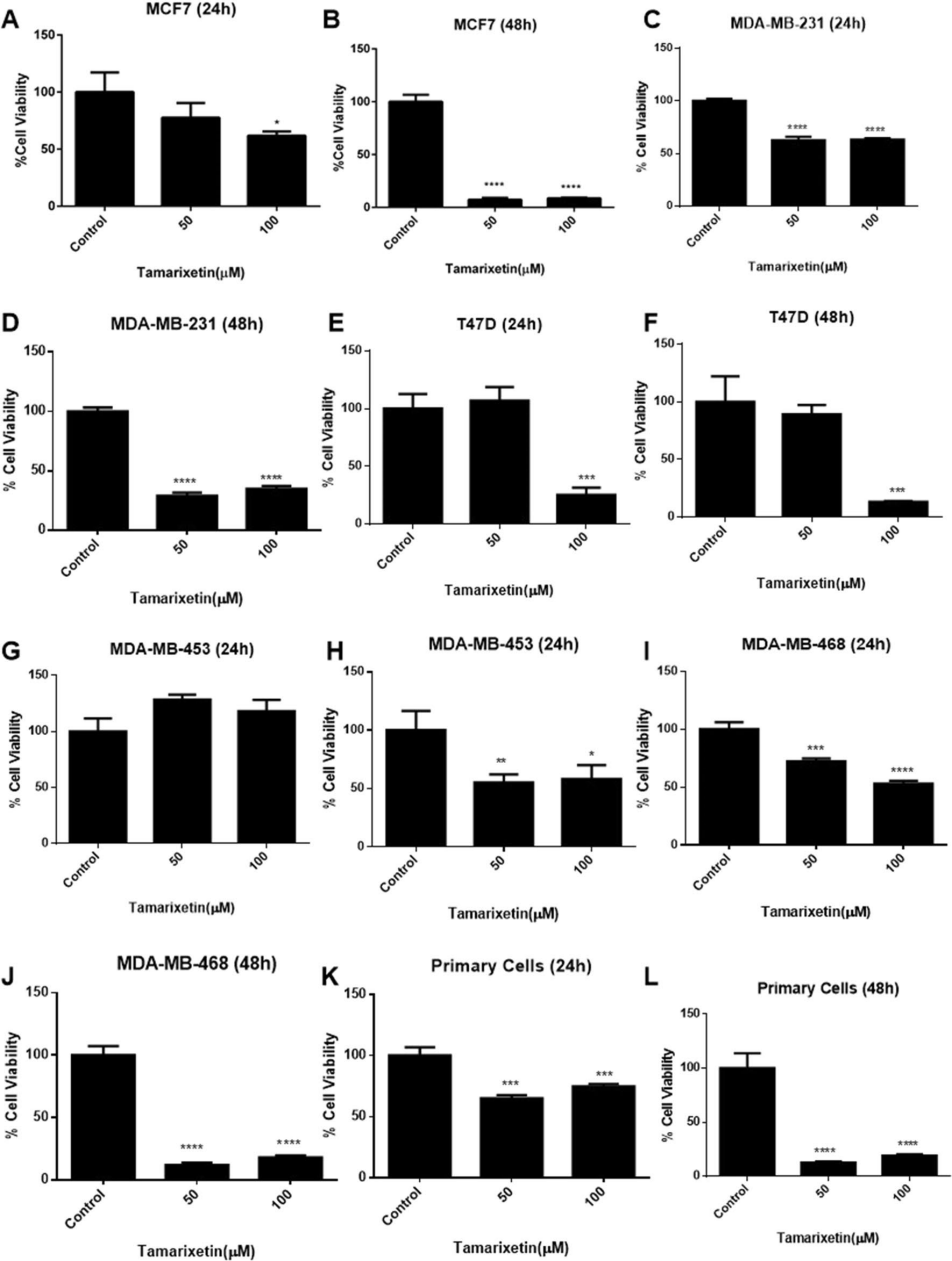
Effect of Tamarixetin on cell proliferation of breast cancer cells. The cells were treated with Tamarixetin for 24 and 48 h and the cell viability was estimated using MTT assay. (**A**) MCF-7 (24 h). (**B**) MCF-7 (48 h). (**C**) MDA-MB-231 (24 h). (**D**) MDA-MB-231 (48 h). (**E**) T47D (24 h). (**F**) T47D (48 h). (**G**) MDA-MB-453 (24 h). (**H**) MDA-MB-453 (48 h). (**I**) MDA-MB-468 (24 h). (**J**) MDA-MB-468 (48 h). (**K**) Breast cancer primary cells (24 h). (**K**) Breast cancer primary cells (48 h).

Network analysis predicted association of Tamarixetin target genes to proliferation, an important hallmark of cancer. Further investigation on the effect of Tamarixetin treatment in breast cancer cells was carried out using MTT assay. The effect of cell proliferation was monitored at 24 h and 48 h after treatment with 50 µM and 100 µM of Tamarixetin (Fig. 6). In MCF-7 cells, 48 h treatment resulted in 92.70% inhibition of cell proliferation at 50 µM Tamarixetin. MDA-MB-231, MDA-MB-468 and MDA-MB-453 cells exhibited 71.02%, 88.14% and 44.97% inhibition of cell proliferation respectively, upon treatment with 50 µM of Tamarixetin for 48 h. Even though the T47D breast cancer cell line did not show any significant reduction in cell proliferation at 50 µM, 86.68% inhibition was observed on treatment with 100 µM of Tamarixetin at 48 h. These results suggest that there are variations in the activity of Tamarixetin depending on the cell lines. However, the majority of the cell lines tested showed a significant reduction in cell viability at 50 µM itself, suggesting the potential of Tamarixetin in inhibiting cell proliferation. These observations confirm our network analysis results showing an association of Tamarixetin target proteins with “Proliferation”, an important hallmark of cancer. The results obtained in breast cancer cell lines were validated in primary cells isolated from the surgical tissue of breast cancer patients, which showed very significant inhibition of cell proliferation (87.20%) on treatment with Tamarixetin (50 µM) for 48 h. Primary cells serve as a superior model to study cancer, since they closely resemble cancer cells in the patient tumor microenvironment. Even though cell lines serve as a feasible cancer model for in vitro studies, they have undergone a series of cell divisions resulting in genetic drift. Therefore, the genetic makeup of the cell line will be different from the patient’s tumor. Since primary cells are isolated directly from patients, their genetic makeup will be identical to that of tumor cells from the cancer patient. Thus, confirmation of observations from immortalised cell lines in primary cells increases the validity and clinical significance of the study and further supports the anti-cancer potential of Tamarixetin in breast cancer cells.

### Tamarixetin inhibits the clonogenic potential of breast cancer cells

**Figure 7:**
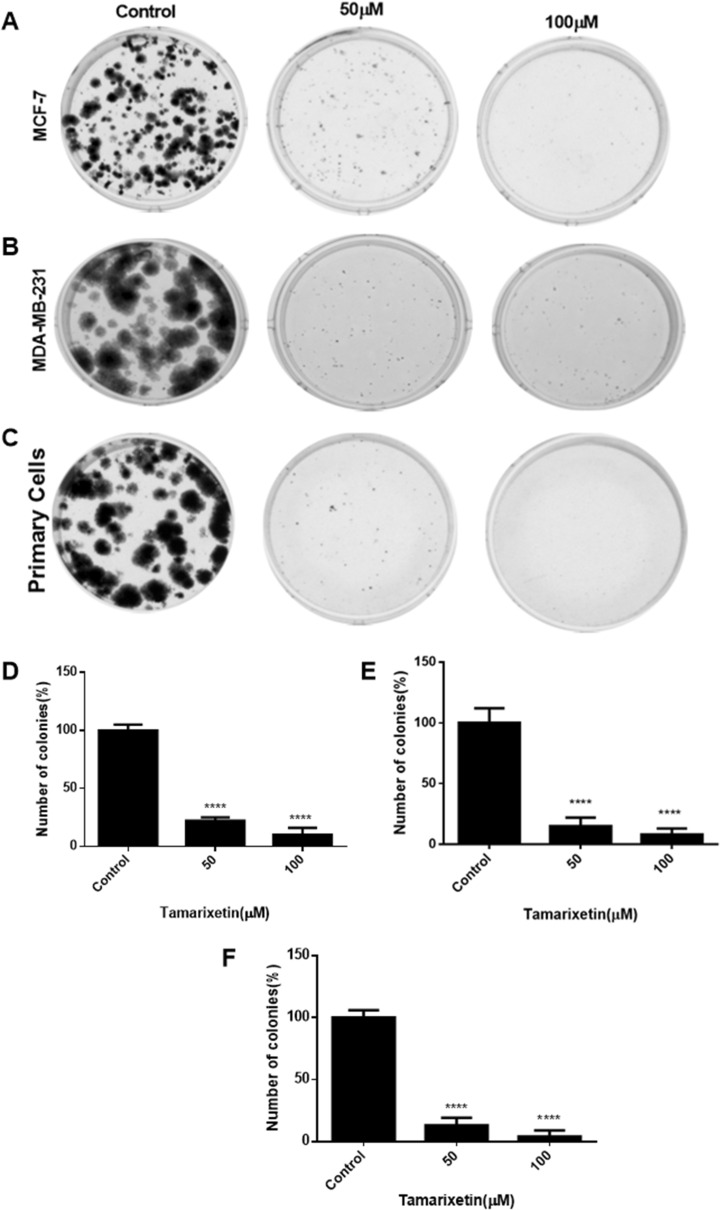
Effect of Tamarixetin on colony formation potential of breast cancer cells. (**A**) MCF-7 cells. (**B**) MDA-MB-231 cells. (**C**) Breast cancer primary cells. (**D**) Quantification of colony-forming potential of MCF-7 cells. (**E**) Quantification of colony-forming potential of MDA-MB-231 cells. (**F**) Quantification of colony-forming potential of breast cancer primary cells.

The clonogenic assay measures the potential of single cells to grow and form a colony of cancer cells. The ability of the cells to undergo unlimited growth depends on cancer hallmarks such as proliferation, immortality and prevention of cell death. Tamarixetin at a concentration of 50 µM and 100 µM abolished the potential of MCF-7 and MDA MB 231 breast cancer cell lines to form independent colonies (Fig. 7). Similar to the observations in breast cancer cell lines, primary cells also showed a drastic reduction in colony-forming potential on treatment with 50 µM and 100 µM of Tamarixetin. These results demonstrate that Tamarixetin is able to effectively inhibit colony formation of breast cancer cell lines as well as primary cells.

### Tamarixetin induces cell death in breast cancer cells

**Figure 8:**
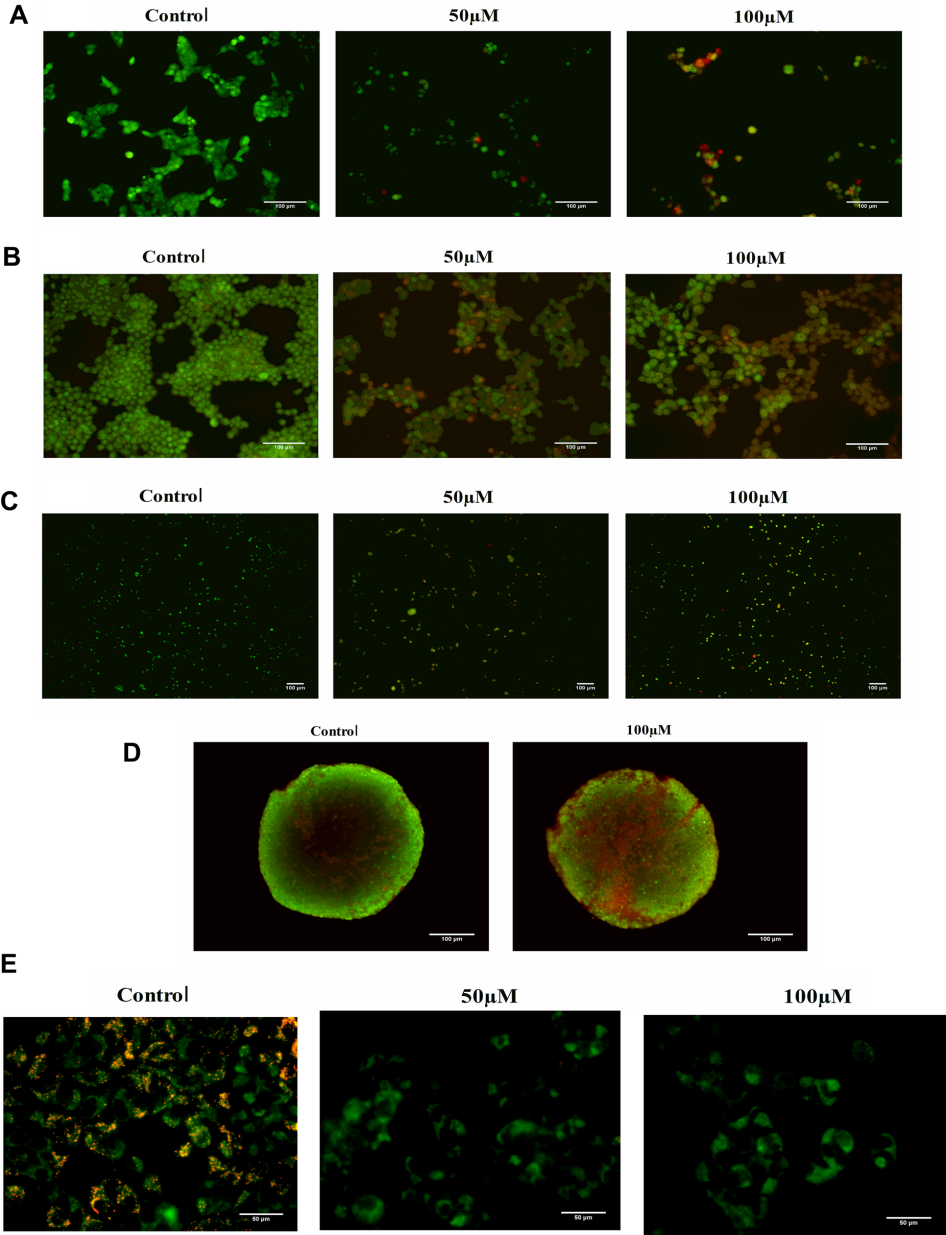
Analysis of cell death. (**A**) MCF-7 cells treated with 50 µM and 100 µM of Tamarixetin for 24 h. Images were taken using the fluorescence microscope at ×100 magnification. (**B**) MCF-7 cells treated with 50 µM and 100 µM of Tamarixetin for 48 h. Images were taken using the fluorescence microscope at ×100 magnification. (**C**) MDA-MB-231 cells treated with 50 µM and 100 µM of Tamarixetin for 48 h. Images were taken using the fluorescence microscope at ×40 magnification. (**D**) Effect of Tamarixetin on MCF-7 spheroids in 3D culture. Spheroids treated with 100 µM of Tamarixetin for 72 h and stained with AO/EB. Images were taken using the fluorescence microscope at ×100 magnification. (**E**) Effect of Tamarixetin on the mitochondrial membrane potential of MCF-7 cells.

Tamarixetin (50 µM and 100 µM) induced cell death in breast cancer cells is reflected by an increase in the number of cells stained with orange fluorescence (Fig. 8A–C) on EB/AO staining. The number of cells undergoing death was increased at 48 h compared to that of 24 h. These observations were further confirmed in a 3D model of tumor spheroids developed from MCF-7 cells which were treated with 100 µM of Tamarixetin for 72 h. AO/EB analysis showed that Tamarixetin is also able to induce cell death in 3D spheroids (Fig. 8D). A decrease in the mitochondrial membrane potential is a signature step in the onset of the apoptotic cascade. Treatment of MCF-7 cells with 100 µM Tamarixetin for 24 h, resulted in a significant reduction in the mitochondrial membrane potential, denoted by loss of red fluorescence (Fig. 8E), which in itself is sufficient for the cancer cells to initiate apoptosis. These observations suggest that Tamarixetin could impair mitochondrial membrane potential leading to cell death.

### Tamarixetin induces cell cycle arrest in MCF-7 cells

**Figure 9:**
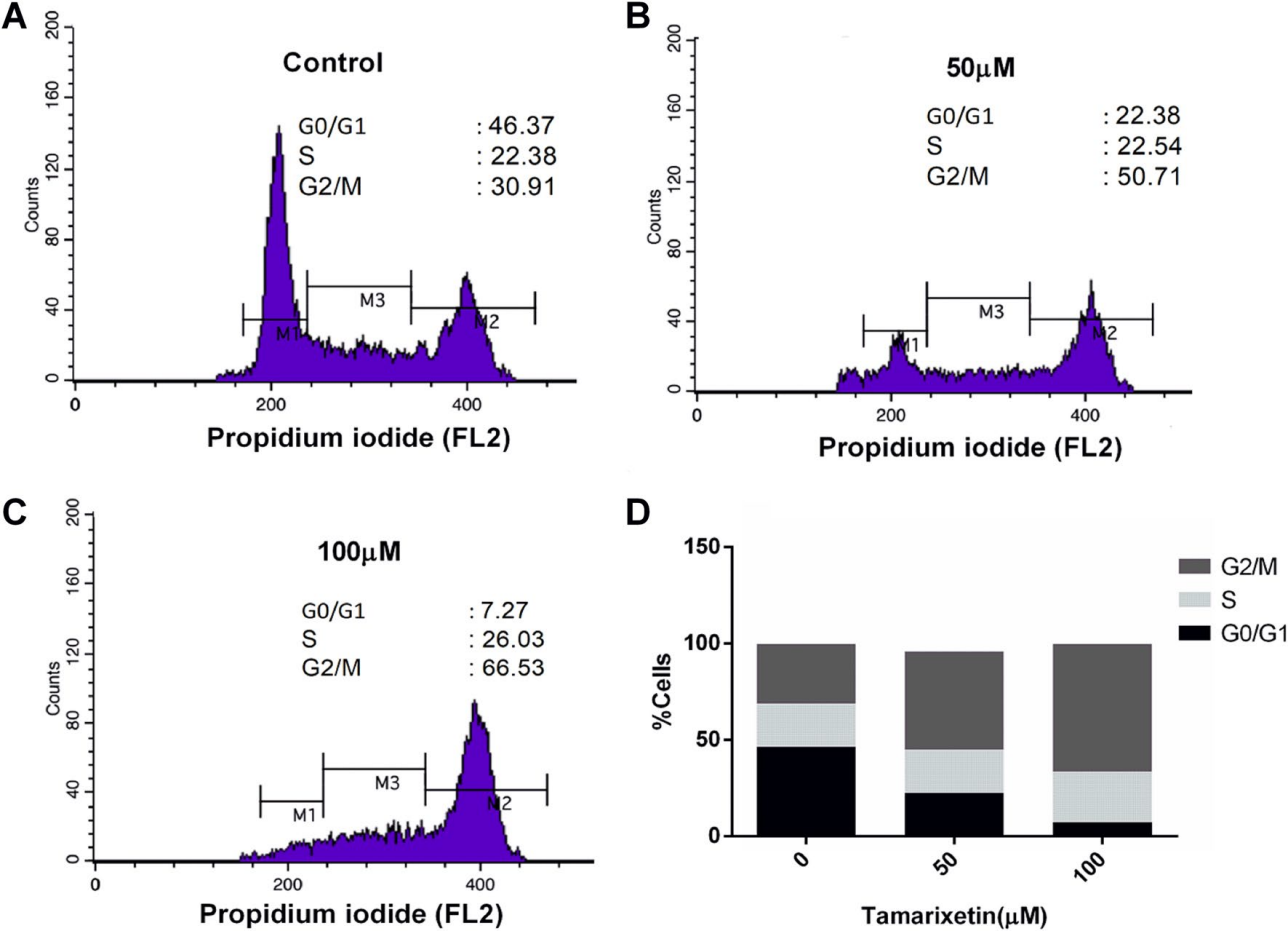
Effect of Tamarixetin on the cell cycle of MCF-7. (**A**) Control cells. (**B**) Cells treated with 50 µM Tamarixetin. (**C**) Cells treated with 100 µM Tamarixetin. (**D**) Graphical representation of the cell cycle profile.

Cell cycle arrest is associated with both inhibition of proliferation as well as induction of apoptosis. Tamarixetin treatment resulted in a distinct dose-dependent alteration in the cell profile, leading to cell cycle arrest at G2/M phase (Fig. 9).

### Tamarixetin inhibits breast cancer cell migration and invasion

**Figure 10:**
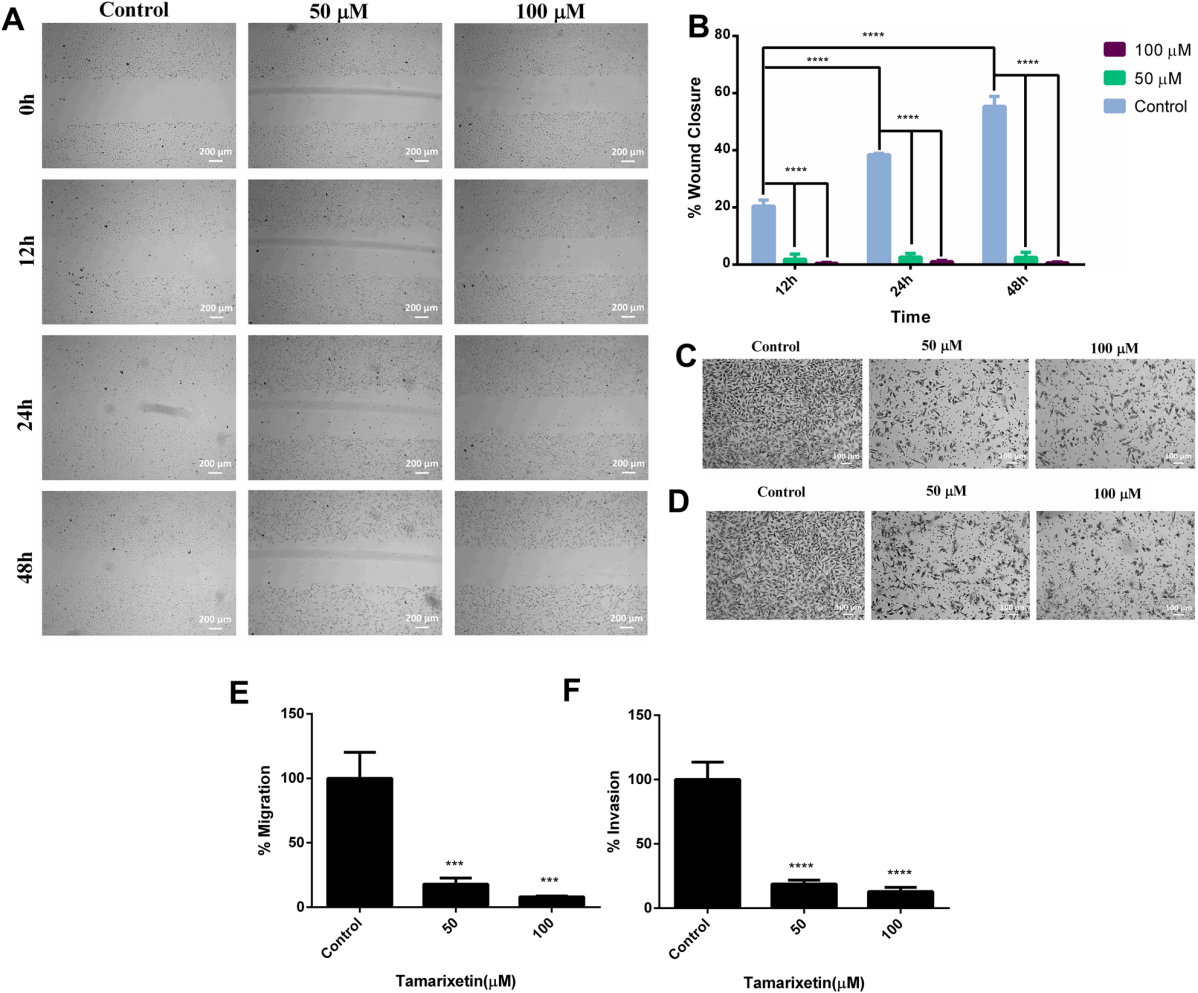
Effect of Tamarixetin on cell migration and invasion of MDA-MB-231 cells. (**A**) Scratch wound healing assay was performed after treatment of Tamarixetin for 48 h. (**B**) Quantification of migration. (**C**) Transwell migration assay was performed after treatment of Tamarixetin for 24 h (**D**) Transwell invasion assay was performed after treatment of Tamarixetin for 24 h. (*E*) Quantification of migration. (**F**) Quantification of invasion.

Migration and invasion leading to metastasis are prominent characteristics of aggressive tumors. The potential of Tamarixetin to inhibit migration was studied using scratch wound healing assay. Treatment with 50 µM and 100 µM of Tamarixetin significantly reduced the migratory potential of MDA-MB-231 cells. In the control cells, 21%, 39% and 55% closure of the scratch wound were observed at 12 h, 24 h and 48 h respectively (Fig. 10A,B). No significant change in the wound area was observed at different time points (12 h, 24 h and 48 h) with 50 µM and 100 µM of Tamarixetin. Transwell chamber assays were performed to confirm the anti-migratory potential of Tamarixetin as well as investigate the anti-invasive potential of Tamarixetin. MDA-MB-231 cells treated with Tamarixetin were seeded on top of the Transwell chamber. For the invasion assay, the chamber used was pre-coated with 200 µg/ml of Matrigel to mimic the biological membrane. Tamarixetin treatment at a concentration of 50 µM and 100 µM drastically abolished both the invasive as well as the migratory potential of MDA-MB-231 cells (Fig. 10C–F).

## Discussion

Tamarixetin is a naturally occurring flavonoid reported to possess anti-cancer, anti-metastatic, anti-inflammatory, cardioprotective and gastroprotective activities. Earlier studies from our laboratory had demonstrated the anti-metastatic potential of Tamarixetin in Fibrosarcoma cells^22^. Tamarixetin was shown to inhibit cell proliferation and induce apoptosis in HepG2 liver cancer cells^26^ and in K562 human leukemia cells^27^. Tamarixetin was also shown to mitigate the efflux transporter breast cancer resistance protein (BCRP/ABCG2)^28^. Even though Tamarixetin showed anti-cancer properties in many types of cancer cells, a detailed mechanism of action of this compound in breast cancer is lacking. Our study provided a possible mechanism by which Tamarixetin could inhibit breast cancer progression. Through reverse docking followed by network pharmacology analysis, we have identified 35 Targets of Tamarixetin. Functional enrichment analysis demonstrated that these proteins play critical roles in development and progression of breast cancer. These observations obtained with in silico studies were further corroborated with in vitro studies confirming the potential of Tamarixetin to induce apoptosis, inhibit cancer cell proliferation, cell cycle progression, migration, invasion and cell survival in primary cells and breast cancer cell lines in 2D as well as in 3D systems.

Various tools such as OncomiR, ENCORI, starBase and GEPIA were used to obtain data from the TCGA database. All the survival analysis and gene expression studies of hub genes and seed genes were performed using the data obtained from the TCGA dataset employing the different tools. The top 3 hub-genes, namely AKT1, ESR1 and HSP90AA1, identified by protein–protein interaction network analysis using Tamarixetin targets, are known to play prominent roles in tumor progression^29–31^. There was a significant induction in expression of both ESR1 and HSP90AA1 observed in breast cancer compared to normal breast tissues (Fig. 3B) and the elevated expression of all three genes were associated with poor survival in breast cancer patients (Fig. 3C). AKT1 plays a critical role in promoting tumor cell survival by preventing cytochrome c release from mitochondria, which is one of the important steps in the initiation of apoptosis^32,33^. AKT1 kinase is constitutively active in many cancers and suppression of AKT activation may attenuate cancer progression^34^. In breast cancer, pAKT expression is negatively correlated with overall as well as disease-free survival of patients^35^. A similar effect of AKT overexpression having an adverse effect on patient survival, was reported in head and neck carcinoma^36^. Consistent with our results, higher expression of HSP90AA1 was reported to unfavourably affect breast cancer patient survival^37^. Even though the Kaplan–Meier survival analysis of ESR1 in breast cancer showed a reduction in patient survival with increased expression, the result was not statistically significant. Similarly, the seed genes SRC and VDR in two functional modules identified in the PPI network were also closely associated with cancer progression. The analysis showed upregulation of these genes in breast tumor tissues and their expression levels to be negatively correlated with patient survival (Fig. 4C). SRC is known to play critical roles in regulating biological functions associated with cancer progression such as cell proliferation, differentiation, cellular migration and angiogenesis^38–40^. Overexpression of SRC is identified in many types of cancers^39–41^. In accordance with our observation, previous studies also reported upregulation of SRC activities in breast cancer tissues compared to normal breast tissues^41–44^. SRC is associated with angiogenesis, cell-adhesion, cell motility, and invasiveness of breast cancer^38^. Furthermore, hypoxia-induced upregulation of VEGF is mediated by the activation of SRC, and its knockdown using RNAi attenuated VEGF expression in breast cancer cells^45,46^. Breast cancer cells showed decreased motility and invasiveness in the presence of SRC inhibitors^47^. Abrogation of SRC in MCF-7 cells resulted in reduced cell migration, attachment, spreading and proliferation^48^. These reports suggest the possibility of treating SRC as a target for breast cancer therapy. SRC might be one of the key targets of Tamarixetin in mitigating breast cancer progression. Even though AKT1 and VDR showed significant negative correlation to patient survival, they did not show major differences in the expression between the normal and tumor tissues. Similar patterns of gene expression were also observed with SRC. Although the difference in gene expression levels are not large enough to show a statistically significant variation, a small change in the gene expression may contribute to elevated levels of protein resulting in increased activity in the signalling cascade. Thus, even small changes in gene expression levels may significantly affect the survival levels of the patients.

Additionally, functional enrichment analysis of target proteins of Tamarixetin indicated that anti-breast cancer properties of the compound are related to different biological processes and pathways associated with cancer. Tamarixetin targets were associated with biological processes such as protein autophosphorylation, negative regulation of apoptotic process and signal transduction—all of which are critical for tumor development and progression. Tamarixetin targets were also associated with Pathways in cancer, Proteoglycans in cancer, PI3K-Akt signaling pathway, Estrogen signaling pathway, Prostate cancer and Rap1 signaling pathway. Dysregulation of PI3K-Akt signaling pathway is seen in many types of cancers including breast cancer^49^. Hyperactivation in this pathway leads to increased cell proliferation, instability in the genome and acquired resistance to anti-cancer therapeutics^50^. This makes PI3K-Akt signaling pathway a potential drug target as well as a prognostic and or diagnostic marker in breast cancer therapy. Our analysis showed that HSP90AA1, PDPK1, NOS3, AKT2, KIT, KDR, AKT1, IGF1, EGFR, FGFR1 and IGF1R proteins in PI3K-Akt signaling pathway are direct targets of Tamarixetin. Estrogen signaling pathway enriched with 9 target proteins (HSP90AA1, SRC, NOS3, AKT2, AKT1, ESR1, MMP9, EGFR, ESR2) is one of the prominent pathways associated with the majority of breast cancers^51^. ESR1 (Estrogen Receptor 1) overexpression is observed in 60–70% of the human breast cancers, and the hyper-activation of the Estrogen signaling pathway provides proliferative advantages, as well as the metastatic potential to the cancer cells. Thus, Estrogen signaling pathway is considered as a potential target for cancer therapy^51^. Target proteins of Tamarixetin such as NOS2, NOS3, AKT2, HMOX1, AKT1, IGF1, EGFR and IGF1R are prominent players in the HIF-1 signaling pathway. HIF-1 signaling cascade is associated with increased tumorigenic potential. Over-expression of HIF-1 signaling pathway leads to increased VEGF levels resulting in increased angiogenesis. Attenuation of this pathway leads to inhibition of tumor growth, angiogenesis and disruption in the energy metabolism of cancer cells^52^. Rap1 signaling pathway plays a very significant role in promoting cell migration in breast cancer cells^53^. Additionally, the Rap1 signaling pathway leads to the induction of MMP 2 and MMP-9 expression, resulting in extracellular matrix degradation, facilitating the invasion of tumor cells^54^. KEGG pathway enrichment analysis showed that 9 targets of Tamarixetin (SRC, AKT2, KIT, KDR, AKT1, IGF1, EGFR, FGFR1 and IGF1R) are playing important roles in this pathway. Aberrant Ras signaling pathway is observed in many tumors including breast cancer. Activation of Ras signaling pathway gives a proliferative advantage to breast cancer cells. Hyperactivation of this pathway leads to the enhanced tumorigenicity and malignant phenotype in breast cancer. Due to the significant role in promoting tumor progression, Ras signaling pathway is considered as a potential target for anti-tumor therapy^55^. KEGG pathway enrichment analysis clearly shows that the target proteins of Tamarixetin are playing significant roles at different stages of cancer progression, indicating the potential anti-cancer mechanisms of the compound. Proteins such as AKT2, KIT, ABL1, KDR, AKT1, IGF1, EGFR, FGFR1 and IGF1R which play key roles in Ras signaling pathway are direct targets of Tamarixetin indicating the anti-cancer potential of Tamarixetin.

Additionally, the target proteins were found to be associated with various hall-marks of cancers such as Proliferation, Glycolysis, Differentiation, Inflammation, DNA-Repair, Angiogenesis, Immortality, Metastasis and Cell death (Fig. 5, Supplementary Fig. S1). In vitro experiments also suggested the potential of Tamarixetin in down regulating cell proliferation, clonogenic potential, cell migration and invasion of breast cancer cells. Additionally, Tamarixetin induced cell cycle arrest as well as cell death in breast cancer cells. In our study, Tamarixetin induced arrest at the G2/M phase of the cell cycle in MCF-7 breast cancer cells. These results are in agreement with an earlier study where Tamarixetin showed a similar trend with G2/M arrest in HL-60 cells^27^. Some of the Targets of Tamarixetin identified in our study are strongly associated with cell cycle progression. Gene ontology analysis of target proteins showed that ERBB4, KIT, RARA, KDR, RARB, IGF1, EGFR, FGFR1 and IGF1R are involved in the ‘positive regulation of cell proliferation’. A previous report showed that the RNAi mediated inhibition of KIT in mouse primary spermatogonial cells induces G2/M phase arrest leading to a reduction in cell proliferation^56^. Inhibition of DNA methyltransferases (DNMTs) in triple-negative breast cancer cells induced G2/M phase arrest with corresponding suppression in RARA levels^57^. Multiple studies have indicated that inhibition of KDR leads to G2/M phase arrest in cancer cells^58,59^ as well as in endothelial cells^60^. Cucurbitacin IIb mediated inhibition of EGFR/mitogen-activated protein kinase (MAPK) pathway leads to G2/M phase cell cycle arrest in lung cancer cells^61^. Thus a combinatorial effect of Tamarixetin mediated inhibition of these genes might be responsible for the observed induction of G2/M phase arrest in MCF-7 cells. The anti-cancer potential of Tamarixetin was validated in both primary breast cancer cells as well as in 3D culture systems which are considered as superior models of cancer progression. These observations suggest that Tamarixetin targets multiple hallmarks of cancer and modulates them at multiple levels leading to the mitigation of breast cancer progression.

The present study did not experimentally investigate the cytotoxic potential of Tamarixetin in normal cells. However, gene expression analysis of target genes showed that the majority of them are overexpressed in cancer tissues compared to normal tissues. Therefore, the chances of cancer cells being affected by Tamarixetin is greater as compared to normal cells. Additionally, to explore the possible toxicity of Tamarixetin, we have performed an in silico study using the pkCSM server^62^ (Supplementary Table S5). The toxicity results from the pkCSM server showed that Tamarixetin does not inhibit hERGI and hERGII and hence is potentially not cardiotoxic. Additionally, it was also predicted to be non-hepatotoxic as well as lacking skin sensitization properties. The maximum tolerated dose in humans was found to be 0.577 log mg/kg/day which is considered as high. These observations suggest that Tamarixetin would not be toxic to normal cells.

Our previous study^22^ showed that Tamarixetin inhibits cell invasion and migration of HT1080 fibrosarcoma cells by inhibiting MMP-9 expression. Similar inhibition of MMP-9 expression in the presence of Tamarixetin was observed in A549 lung cancer cells as well as A375 melanoma cells. Similarly, in this study too, MMP-9 was predicted to be a critical target of Tamarixetin and furthermore the experimental analysis demonstrated Tamarixetin to inhibit breast cancer cell migration as well as invasion. Similarly, EGFR is a key player in cell proliferation^63^ and in vitro studies clearly demonstrate an inhibition of cell proliferation, suggesting an interaction between the two. Thus the in silico predictions seem to correlate well with experimental analysis, which gives an insight into the mechanism of action of the compound. A detailed expression study of the target proteins and their associated genes would provide further clarity. This study therefore forms the basis for the further in silico as well as in vitro analysis.

In conclusion, the present study identified the potential of Tamarixetin to target 35 proteins associated with breast cancer, many of which play critical roles in the pathways connected with neoplastic signaling. These observations were further confirmed and validated by in vitro experiments in breast cancer cell lines, as well as in primary cells, which closely represent the tumor microenvironment, emphasising the potential of Tamarixetin to mitigate breast cancer progression. The effect of Tamarixetin on normal cells was not experimentally checked in the current study. However, toxicity analysis of Tamarixetin in silico showed that the compound is non-toxic in nature. Lack of experimental analysis of gene expression profiles of target genes in breast cancer cell lines is another limitation in the current study. However, the combination of reverse docking to identify potential targets of Tamarixetin followed by network pharmacology approach and in vitro experimental analysis provide a strong basis for the proposed anti-cancer mechanism of Tamarixetin. Another strength of the study is the use of primary cells as well as 2D and 3D culture systems to validate anti-neoplastic properties of the compound. Thus, the present study provides an insight into the mechanism of action of Tamarixetin in breast cancer and provides compelling evidence for further therapeutic exploration of this compound or its derivatives to target pathways involved in breast cancer.

## Materials and methods

### Cell line culture

Tamarixetin was commercially obtained from Extrasynthese, France (Purity ≥ 99%). Human breast cancer cell lines (MDA-MB-231, MDA-MB-468, MDA-MB-453, T47D and MCF-7) cells were obtained from ATCC, USA through National Centre for Cell Science, Pune Maharashtra, India. Cells were cultured in DMEM media (Sigma Aldrich, USA), supplemented with 10% FBS (GIBCO, USA). All media compositions contained 100 mg/ml Penicillin, 100 mg/ml Streptomycin and 0.5 µg/ml Amphotericin B (Sigma Aldrich, USA). Primary cells were cultured, as previously described^64^.

### Identification of anti-cancer targets of Tamarixetin

Anti-cancer targets of Tamarixetin were identified as described earlier^13^. Database of gene-disease associations (DisGeNET) was used to identify genes associated with breast cancer (https://www.disgenet.org/). Gene disease association with strong evidence (GDA score of ≥ 0.3) was selected for further analysis^65^. Reverse docking was performed using PharmMapper (http://www.lilab-ecust.cn/pharmmapper/), which can identify human protein targets of compounds using pharmacophore mapping^66,67^. Following parameters were used while performing reverse docking using PharmMapper. The option for "Energy Minimization" present in "Conformation Generation" was set to “Yes”. The option "Human protein targets only" was selected under option "Pharmacophore Mapping". The option of "Perform GA Match" was set to “Yes” and all other options were set as default. The breast cancer associated genes identified from DisGeNET, which was present in the target list obtained from reverse docking, was considered as the target of the Tamarixetin in breast cancer.

### Protein–protein interaction (PPI) network

Protein–protein interaction network of Tamarixetin target proteins was created using the STRING database (https://string-db.org)^68^. The ‘minimum required interaction score” was set to “high confidence (0.700)” and all other options in the “Settings” were set as default. The PPI network was constructed and was visualized in Cytoscape^69^. NetworkAnalyzer^70^ in Cytoscape was used for the topological analysis of the PPI network.

### Identification of functional modules and hub genes

Functional modules (highly interconnected regions) in the network were identified using Cytoscape plugin MCODE^71^. The gene in the functional module with highest MCODE score was selected as the seed gene of the subnetwork. The hub genes were identified using Cytoscape plugin CytoHubba^72^. Algorithms based on Maximal Clique Centrality (MCC) were used to identify the important nodes in the network.

### Gene ontology (GO) and pathway enrichment analysis

The Database for Annotation, Visualization and Integrated Discovery (DAVID) (http://david.ncifcrf.gov/)^73^ was used for Gene ontology (GO) and Kyoto Encyclopedia of Genes and Genomes (KEGG)^74^ pathway enrichment analysis. The enriched pathways as well as gene ontologies with a False Discovery Rate, FDR < 0.05 was considered as significant. The target proteins were categorized into molecular function, cellular components and biological processes ontologies. KEGG pathways analysis was performed to identify pathways associated with target genes.

### Association of target genes with cancer hallmarks

CancerGeneNet server (https://signor.uniroma2.it/CancerGeneNet)^25^ was used to identify the association of target genes involved in various hallmarks of cancers. The target genes list was uploaded into the server to obtain the association of target genes to cancer hallmarks.

### Accession and analysis of TCGA and GTEx database

The Cancer Genome Atlas (TCGA) is a project focused on unravelling cancer associated genomics information, using high throughput genome analysis technologies, coupled with bioinformatics analysis to facilitate better understanding of the disease. Currently TCGA data consists of genomics information of around 20,000 cancer samples, along with their normal samples belonging to over 33 types of cancer. This information is available in public domain and is freely accessible for further analysis using Bioinformatics tools aiding cancer research. Similarly, Genotype-Tissue Expression (GTEx)^75^, is a project focusing on collecting tissue specific genomics information across around 1000 individuals. The data can be accessed from the GTEx portal for further analysis. Several Bioinformatics tools are available for accessing and analysing both TCGA and GTEx databases. OncomiR (www.oncomir.org) and ENCORI: The Encyclopedia of RNA Interactomes—starBase (starBasestarbase.sysu.edu.cn), GEPIA (gepia.cancer-pku.cn)^76^, were used for differential expression analysis of genes and miRNAs in normal and tumor tissues from TCGA and GTEx database. GEPIA was used for the analysis of correlation between genes.

### Kaplan–Meier survival curves analysis

Data from the TCGA database was accessed using Kaplan Meier plotter (https://kmplot.com/analysis)^77^ to perform survival analysis. Additionally, microarray datasets present in Kaplan Meier plotter were also used for calculating gene/miRNA expression and its correlation to survival of the patients.

### MTT assay for cell viability

Cell viability was analyzed using MTT assay (3-(4,5-dimethylthiazol-2-yl)-2,5-diphenyltetrazolium bromide). Cells (5 × 10^3^ cells) were seeded onto 96 well plates and incubated at 37 °C for 24 h. The cells were treated with different concentrations of compounds. After incubation in the presence of compound, 10 µl of MTT reagent (Sigma Aldrich, USA) at a concentration of 5 mg/ml was added. After 3 h of incubation, the Formazan crystals generated were dissolved in 100 µl of DMSO (Sigma Aldrich, USA). Absorbance at 590 nm and 620 nm was read in the Microplate Reader (BioTek Instruments Inc., USA) and used for the calculation of cell viability. Cell viability was calculated using the following formula,

$$\% \mathrm{Cell} \, \mathrm{Viability}=(\mathrm{Mean} \, \mathrm{OD} \, \mathrm{of} \, \mathrm{the} \, \mathrm{treated} \, \mathrm{cells}/\mathrm{Mean} \, \mathrm{OD} \, \mathrm{of} \, \mathrm{ the} \, \mathrm{ control} \, \mathrm{cells}) \times 100$$

### In vitro scratch wound healing assay

To determine the role of Tamarixetin on MDA-MB-231 cell migration, scratch wound healing assay was performed as described^78^. MDA-MB-231 cells were pre-treated for 12 h with Tamarixetin, washed with PBS, and a linear wound was created using a micropipette tip. Reference points to take the image were marked with a pen on the exterior surface of the 6 well plate. The debris was washed out using PBS and DMEM serum-free medium. Cells were incubated with different concentrations of Tamarixetin. Equal amounts of DMSO were added to Control wells. After 12 h of incubation at 37 °C, the inverted bright field image at 50X magnification was taken in a ZEISS Inverted phase contrast microscope. Images were acquired using Gen5 2.01 Software and analysis was performed using ImageJ 1.49^79^.

### Transwell Matrigel invasion assay

MDA-MB-231 cells were treated with Tamarixetin for 12 h, harvested, and seeded (2.5 × 10^4^ cells) onto 24 well Transwell inserts with 0.8 μm pore size (BD Biosciences, USA), pre-coated with Matrigel (BD Biosciences, USA) at a concentration of 200 µg/ml and incubated at 4 °C overnight. The cells were allowed to migrate for 24 h in response to 10% FBS, in the presence of Tamarixetin. The migrating cells were fixed with 5% glutaraldehyde, stained with Crystal Violet and observed under an Inverted microscope (Olympus). The number of cells obtained in five different fields at 100× and 200× magnification were counted manually. The experiments were performed in triplicates.

### Clonogenic assay

The Clonogenic assay was performed to analyze the colony forming capacity of individual cells. MDA-MB-231, MCF-7 and primary cells (500 cells/ well) were seeded onto 12 well plates and kept undisturbed for 24 h. Cells were treated with Tamarixetin and incubated at 37 °C. On the 10th day of treatment, the cells were fixed with 100% ice-cold methanol for 15 min, followed by staining with 1% Crystal violet solution for 1 h. Images were taken using Gel Doc (Bio-Rad Laboratories, USA) imaging system. Crystal violet stain was extracted with 20% acetic acid and quantified using Microplate Reader (BioTek Instruments, USA) at 590 nm.

### 3D spheroid culture

Liquid overlay method was employed for spheroid generation^80^. 96 well plates were coated with 1.5% agarose. 200 µl of MCF-7 cells (2.5 × 10^4^ cells/ml) were seeded on top of the agarose. Plates were centrifuged for 5 min at 1000 g and incubated at 37 °C for 3 days. Since PMA was observed to disrupt the spheroids, all these experiments were conducted in the absence of PMA.

### Cell cycle analysis

Cells (6 × 10^4^ for 24 h treatment and 3 × 10^4^ for 48 treatment) were seeded in 6 well plates and incubated at 37 °C for 24 h, in 5% CO_2_ incubators. Cells were treated for either 24 h or 48 h with Tamarixetin. The cells were lysed using a cell lysis buffer containing 0.05 mg/ml propidium iodide (PI) and RNaseA for 15 min at 25 °C in a dark room. The analysis was performed in BD FACSCalibur™ using the FL2 channel for PI fluorescence detection. BD DNA QC Particles kit was used for QC analysis. BD CellQuest Pro software was used for the acquisition and analysis of the data.

### Apoptosis studies

The staining stock solution was prepared by mixing 400 µg/ml acridine orange (AO) and 100 µg/ml ethidium bromide (EB) in PBS. 1 µl of staining stock solution was added to 999 µl of PBS to make the working staining solution. The cells were treated with 100 µl of working staining solution for 5 min and imaged using fluorescence microscope at 40× and 100× magnification.

### Mitochondrial membrane potential analysis

Loss of mitochondrial membrane potential was analysed using JC-1 staining, following manufacturer’s protocol (Thermo Fisher Scientific). After treatment with Tamarixetin, the cells were washed with PBS and incubated with JC-1 dye, at a concentration of 100 µg/ml in cell culture media at 37 °C for 15 min. Cells were washed and imaged through Fluorescent microscope at 100×, 200× and 400× magnifications. Intact mitochondria exhibit red JC-1 aggregates, while mitochondria with low membrane potential fluorescence in green.

### Statistical analysis

Statistical analysis was carried out using Prism (GraphPad Software Inc., San Diego, CA). One-way analysis of variance (one way ANNOVA) or Student's t test was performed on the values, expressed as the mean ± standard deviation (SD) of at-least 3 independent experiments. A P value less than 0.05 was defined as significant.

## Supplementary Information

Supplementary Information.
