# Supplementary material for: Systematic understanding of anti-tumor mechanisms of Tamarixetin through network and experimental analyses
Source: Sci Rep. 2022 Mar 10;12:3966. doi: 10.1038/s41598-022-07087-6 (PMC8913656; doi:10.1038/s41598-022-07087-6)
Supplement: Supplementary file 1 — Supplementary Information. [file 41598_2022_7087_MOESM1_ESM.pdf]

Supplementary Materials

Systematic understanding of anti-tumor mechanisms of Tamarixetin through network and experimental analyses

Sanu K. Shaji, Drishya G., Damu Sunilkumar, Prashanth Suravajhala, Geetha B. Kumar*, Bipin G. Nair*

Supplementary Table S1. Significantly enriched GO terms in the biological process category

Term	Count	FDR	Genes
GO:0046777~protein autophosphorylation	12	1.71E-11	ERBB4, PDPK1, SRC, KIT, ABL1, PIM1, KDR, AKT1, EPHB4, EGFR, FGFR1, IGF1R
GO:0043066~negative regulation of apoptotic process	15	3.10E-11	NQO1, SRC, GSTP1, IGF1, MIF, SOD2, MMP9, EGFR, IGF1R, ERBB4, PIM1, KDR, RARA, AKT1, RARB
GO:0018108~peptidyl-tyrosine phosphorylation	9	1.51E-07	HSP90AA1, ERBB4, SRC, KIT, ABL1, KDR, EPHB4, EGFR, FGFR1
GO:0048015~phosphatidylinositol-mediated signaling	8	2.96E-07	ERBB4, PDPK1, KIT, AKT1, IGF1, EGFR, FGFR1, IGF1R
GO:0007165~signal transduction	14	2.42E-05	HSP90AA1, SRC, VDR, IGF1, ESR1, EGFR, ESR2, IGF1R, ERBB4, AKT2, KIT, RARA, AKT1, RARB
GO:0001934~positive regulation of protein phosphorylation	7	2.42E-05	ERBB4, AKT2, ABL1, KDR, AKT1, MMP9, EGFR
GO:0032869~cellular response to insulin stimulus	6	4.93E-05	PDPK1, SRC, AKT2, GSTP1, AKT1, APRT
GO:0038128~ERBB2 signaling pathway	5	9.53E-05	HSP90AA1, ERBB4, SRC, AKT1, EGFR

GO:0038083~peptidyl-tyrosine autophosphorylation	5	9.61E-05	ERBB4, SRC, ABL1, KDR, IGF1R
GO:0070374~positive regulation of ERK1 and ERK2 cascade	7	9.61E-05	NQO2, ERBB4, SRC, ABL1, KDR, MIF, EGFR
GO:0045429~positive regulation of nitric oxide biosynthetic process	5	1.15E-04	HSP90AA1, AKT1, SOD2, ESR1, EGFR
GO:0006809~nitric oxide biosynthetic process	4	1.38E-04	NQO1, NOS2, NOS3, AKT1
GO:0008284~positive regulation of cell proliferation	9	1.90E-04	ERBB4, KIT, RARA, KDR, RARB, IGF1, EGFR, FGFR1, IGF1R
GO:0048146~positive regulation of fibroblast proliferation	5	2.28E-04	ABL1, IGF1, MIF, ESR1, EGFR
GO:0043401~steroid hormone mediated signaling pathway	5	2.65E-04	RXRB, VDR, RARA, RARB, ESR1
GO:0043406~positive regulation of MAP kinase activity	5	2.85E-04	SRC, KIT, MIF, EGFR, FGFR1
GO:0014068~positive regulation of phosphatidylinositol 3-kinase signaling	5	3.96E-04	ERBB4, KIT, KDR, IGF1, FGFR1
GO:0006367~transcription initiation from RNA polymerase II promoter	6	5.51E-04	RXRB, VDR, RARA, RARB, ESR1, ESR2
GO:0014066~regulation of phosphatidylinositol 3-kinase signaling	5	7.32E-04	ERBB4, KIT, AKT1, EGFR, FGFR1
GO:0050999~regulation of nitric-oxide synthase activity	4	7.39E-04	HSP90AA1, NOS3, AKT1, EGFR
GO:0045909~positive regulation of vasodilation	4	9.85E-04	NOS2, NOS3, HMOX1, EGFR
GO:0030335~positive regulation of cell migration	6	0.001132	AKT2, KIT, KDR, IGF1, EGFR, IGF1R

GO:0071364~cellular response to epidermal growth factor stimulus	4	0.001315	PDPK1, GSTP1, AKT1, EGFR
GO:0007169~transmembrane receptor protein tyrosine kinase signaling pathway	5	0.001315	ERBB4, KIT, KDR, EGFR, IGF1R
GO:0071222~cellular response to lipopolysaccharide	5	0.002389	NOS2, SRC, GSTP1, ABL1, RARA
GO:0008283~cell proliferation	7	0.002459	ERBB4, SRC, PIM1, AKT1, IGF1, MIF, EGFR
GO:0031659~positive regulation of cyclin-dependent protein serine/threonine kinase activity involved in G1/S transition of mitotic cell cycle	3	0.00317	PIM1, AKT1, EGFR
GO:0009408~response to heat	4	0.003407	HSP90AA1, NOS3, AKT1, IGF1
GO:0010907~positive regulation of glucose metabolic process	3	0.003663	SRC, AKT2, AKT1
GO:0010863~positive regulation of phospholipase C activity	3	0.003663	KIT, ESR1, FGFR1
GO:0007173~epidermal growth factor receptor signaling pathway	4	0.004878	PDPK1, SRC, ABL1, EGFR
GO:0048661~positive regulation of smooth muscle cell proliferation	4	0.005801	HMOX1, AKT1, IGF1, EGFR
GO:0019430~removal of superoxide radicals	3	0.006082	NQO1, NOS3, SOD2
GO:0060644~mammary gland epithelial cell differentiation	3	0.006769	ERBB4, AKT2, AKT1
GO:0031641~regulation of myelination	3	0.006769	RARA, RARB, AKT1
GO:0043065~positive regulation of apoptotic process	6	0.006885	ERBB4, SRC, ABL1, HMOX1, RARB, AKT1

GO:0048009~insulin-like growth factor receptor signaling pathway	3	0.007461	AKT1, IGF1, IGF1R
GO:0045725~positive regulation of glycogen biosynthetic process	3	0.008371	AKT2, AKT1, IGF1
GO:0042523~positive regulation of tyrosine phosphorylation of Stat5 protein	3	0.010538	ERBB4, KIT, IGF1
GO:0043410~positive regulation of MAPK cascade	4	0.011096	KIT, KDR, IGF1, FGFR1
GO:0048384~retinoic acid receptor signaling pathway	3	0.011096	RXRB, RARA, RARB
GO:0050731~positive regulation of peptidyl-tyrosine phosphorylation	4	0.011096	SRC, ABL1, IGF1, MIF
GO:0030520~intracellular estrogen receptor signaling pathway	3	0.014351	SRC, ESR1, ESR2
GO:0032355~response to estradiol	4	0.014351	NQO1, GSTP1, RARA, ESR1
GO:0046854~phosphatidylinositol phosphorylation	4	0.015418	ERBB4, KIT, EGFR, FGFR1
GO:0001525~angiogenesis	5	0.017283	NOS3, KDR, HMOX1, EPHB4, FGFR1
GO:0008285~negative regulation of cell proliferation	6	0.018549	ERBB4, VDR, NOS3, RARA, RARB, SOD2
GO:0035556~intracellular signal transduction	6	0.019419	PDPK1, SRC, AKT2, KIT, HMOX1, AKT1
GO:0051091~positive regulation of sequence-specific DNA binding transcription factor activity	4	0.019419	KIT, AKT1, ESR1, ESR2
GO:0032148~activation of protein kinase B activity	3	0.019419	PDPK1, SRC, IGF1

GO:0006979~response to oxidative stress	4	0.021426	ABL1, HMOX1, AKT1, EGFR
GO:2000145~regulation of cell motility	3	0.021662	ERBB4, ABL1, EGFR
GO:0046326~positive regulation of glucose import	3	0.024397	AKT2, AKT1, IGF1
GO:0043552~positive regulation of phosphatidylinositol 3-kinase activity	3	0.025564	ERBB4, SRC, KIT
GO:0045907~positive regulation of vasoconstriction	3	0.026738	ABL1, AKT1, EGFR
GO:0018105~peptidyl-serine phosphorylation	4	0.028141	PDPK1, SRC, AKT2, AKT1
GO:0010629~negative regulation of gene expression	4	0.034476	NOS2, AKT1, MIF, ESR1
GO:0001501~skeletal system development	4	0.034476	VDR, IGF1, MMP9, FGFR1
GO:0001657~ureteric bud development	3	0.034476	RARA, RARB, FGFR1
GO:2001237~negative regulation of extrinsic apoptotic signaling pathway	3	0.034476	SRC, GSTP1, IGF1
GO:0043124~negative regulation of I-kappaB kinase/NF-kappaB signaling	3	0.03753	GSTP1, ABL1, ESR1
GO:0045944~positive regulation of transcription from RNA polymerase II promoter	8	0.038048	RXRB, VDR, RARA, RARB, AKT1, IGF1, ESR1, EGFR
GO:0045740~positive regulation of DNA replication	3	0.039386	IGF1, EGFR, IGF1R
GO:0007595~lactation	3	0.039386	ERBB4, VDR, APRT
GO:0043525~positive regulation of neuron apoptotic process	3	0.04062	NQO1, NQO2, ABL1

GO:0045893~positive regulation of transcription, DNA-templated	6	0.041624	ERBB4, SRC, RARA, IGF1, ESR1, ESR2
GO:0014806~smooth muscle hyperplasia	2	0.046247	NOS3, HMOX1
GO:0008630~intrinsic apoptotic signaling pathway in response to DNA damage	3	0.046247	ABL1, HMOX1, SOD2

Supplementary Table S2. Significantly enriched GO terms in molecular functions category

Term	Count	FDR	Genes
GO:0004713~protein tyrosine kinase activity	9	3.23E-08	HSP90AA1, ERBB4, SRC, KIT, ABL1, KDR, EGFR, FGFR1, IGF1R
GO:0004714~transmembrane receptor protein tyrosine kinase activity	6	1.03E-06	ERBB4, KIT, KDR, EPHB4, EGFR, IGF1R
GO:0003707~steroid hormone receptor activity	6	5.08E-06	RXRB, VDR, RARA, RARB, ESR1, ESR2
GO:0030235~nitric-oxide synthase regulator activity	4	1.83E-05	HSP90AA1, AKT1, ESR1, EGFR
GO:0004716~receptor signaling protein tyrosine kinase activity	4	3.12E-05	ERBB4, KIT, KDR, EGFR
GO:0005524~ATP binding	14	9.03E-05	HSP90AA1, PDPK1, SRC, EGFR, IGF1R, ERBB4, AKT2, KIT, PIM1, KDR, ABL1, AKT1, EPHB4, FGFR1
GO:0005515~protein binding	31	2.17E-04	SRC, GSTP1, EGFR, IGF1R, RXRB, CASP7, ERBB4, AKT2, KDR, ABL1, PIM1, AKT1, HMOX1, EPHB4, NQO1, NQO2, HSP90AA1,

			NOS2, NOS3, PDPK1, VDR, MMP3, IGF1, MIF, MMP9, ESR1, ESR2, KIT, RARA, ALDOA, FGFR1
GO:0042802~identical protein binding	10	2.70E-04	NQO1, HSP90AA1, AKT1, ALDOA, SOD2, ESR1, MMP9, EGFR, FGFR1, IGF1R
GO:0005158~insulin receptor binding	4	6.31E-04	PDPK1, SRC, IGF1, IGF1R
GO:0019899~enzyme binding	7	8.36E-04	SRC, RARA, HMOX1, AKT1, ESR1, EGFR, ESR2
GO:0020037~heme binding	5	0.00255	NOS2, SRC, NOS3, HMOX1, CYP19A1
GO:0046934~phosphatidylinositol-4,5-bisphosphate 3-kinase activity	4	0.003818	ERBB4, KIT, EGFR, FGFR1
GO:0004672~protein kinase activity	6	0.009594	PDPK1, SRC, AKT2, ABL1, AKT1, EGFR
GO:0005178~integrin binding	4	0.01529	SRC, KDR, IGF1, EGFR
GO:0005088~Ras guanyl-nucleotide exchange factor activity	4	0.01854	ERBB4, KIT, EGFR, FGFR1
GO:0004879~RNA polymerase II transcription factor activity, ligand-activated sequence-specific DNA binding	3	0.026141	RXRB, ESR1, ESR2
GO:0043565~sequence-specific DNA binding	6	0.03683	RXRB, VDR, RARA, RARB, ESR1, ESR2
GO:0005080~protein kinase C binding	3	0.037277	SRC, ABL1, AKT1
GO:0038052~RNA polymerase II transcription factor activity,	2	0.037277	ESR1, ESR2

estrogen-activated sequence-specific DNA binding			
GO:0030145~manganese ion binding	3	0.039953	ABL1, PIM1, SOD2
GO:0005102~receptor binding	5	0.04453	NOS2, SRC, ABL1, RARA, MIF
GO:0004517~nitric-oxide synthase activity	2	0.048244	NOS2, NOS3

Supplementary Table S3. Significantly enriched GO terms in cellular compartments category

Term	Count	FDR	Genes
GO:0005654~nucleoplasm	16	0.002284	NQO2, HSP90AA1, PDPK1, VDR, MIF, ESR1, ESR2, APRT, RXRB, CASP7, ERBB4, AKT2, ABL1, RARA, AKT1, RARB
GO:0043235~receptor complex	5	0.002284	ERBB4, VDR, EGFR, FGFR1, IGF1R
GO:0005634~nucleus	22	0.002284	HSP90AA1, NOS2, NOS3, SRC, VDR, GSTP1, ESR1, EGFR, ESR2, RXRB, CASP7, ERBB4, AKT2, PIM1, KDR, ABL1, RARA, AKT1, HMOX1, RARB, ALDOA, FGFR1
GO:0005829~cytosol	17	0.002284	NQO1, HSP90AA1, NOS2, NOS3, PDPK1, SRC, GSTP1, APRT, CASP7, ERBB4, AKT2, ABL1, AKT1, HMOX1, ALDOA, EPHB4, FGFR1

GO:0005886~plasma membrane	18	0.007135	HSP90AA1, NOS3, PDPK1, SRC, GSTP1, IGF1, ESR1, EGFR, IGF1R, ERBB4, AKT2, KIT, PIM1, KDR, AKT1, HMOX1, EPHB4, FGFR1
GO:0005576~extracellular region	11	0.007158	HSP90AA1, ERBB4, MMP3, KDR, IGF1, MIF, ALDOA, MMP9, EPHB4, ESR2, FGFR1
GO:0048471~perinuclear region of cytoplasm	7	0.010922	HSP90AA1, NOS2, SRC, ABL1, RARA, HMOX1, EGFR
GO:0005615~extracellular space	9	0.029349	GSTP1, KIT, MMP3, HMOX1, IGF1, MIF, ALDOA, MMP9, EGFR

Supplementary Table 4. Significantly enriched pathways in the KEGG database

**Term**	**Count**	**FDR**	**Genes**
hsa05200:Pathways in cancer	15	4.96E-08	HSP90AA1, NOS2, GSTP1, IGF1, MMP9, EGFR, IGF1R, RXRB, AKT2, KIT, ABL1, RARA, AKT1, RARB, FGFR1
hsa05205:Proteoglycans in cancer	12	4.96E-08	ERBB4, PDPK1, SRC, AKT2, KDR, AKT1, IGF1, ESR1, MMP9, EGFR, FGFR1, IGF1R
hsa04915:Estrogen signaling pathway	9	4.47E-07	HSP90AA1, SRC, NOS3, AKT2, AKT1, ESR1, MMP9, EGFR, ESR2

hsa05215:Prostate cancer	8	3.38E-06	HSP90AA1, PDPK1, AKT2, AKT1, IGF1, EGFR, FGFR1, IGF1R
hsa04066:HIF-1 signaling pathway	8	4.94E-06	NOS2, NOS3, AKT2, HMOX1, AKT1, IGF1, EGFR, IGF1R
hsa04151:PI3K-Akt signaling pathway	11	4.55E-05	HSP90AA1, PDPK1, NOS3, AKT2, KIT, KDR, AKT1, IGF1, EGFR, FGFR1, IGF1R
hsa04015:Rap1 signaling pathway	9	6.38E-05	SRC, AKT2, KIT, KDR, AKT1, IGF1, EGFR, FGFR1, IGF1R
hsa05223:Non-small cell lung cancer	6	6.99E-05	RXRB, PDPK1, AKT2, RARB, AKT1, EGFR
hsa04014:Ras signaling pathway	9	8.55E-05	AKT2, KIT, ABL1, KDR, AKT1, IGF1, EGFR, FGFR1, IGF1R
hsa05218:Melanoma	6	1.81E-04	AKT2, AKT1, IGF1, EGFR, FGFR1, IGF1R
hsa04068:FoxO signaling pathway	7	3.00E-04	PDPK1, AKT2, AKT1, IGF1, SOD2, EGFR, IGF1R
hsa04510:Focal adhesion	8	3.36E-04	PDPK1, SRC, AKT2, KDR, AKT1, IGF1, EGFR, IGF1R
hsa04012:ErbB signaling pathway	6	3.74E-04	ERBB4, SRC, AKT2, ABL1, AKT1, EGFR
hsa05221:Acute myeloid leukemia	5	9.08E-04	AKT2, KIT, RARA, PIM1, AKT1
hsa04370:VEGF signaling pathway	5	0.001148	SRC, NOS3, AKT2, KDR, AKT1
hsa04919:Thyroid hormone signaling pathway	6	0.001148	RXRB, PDPK1, SRC, AKT2, AKT1, ESR1
hsa05230:Central carbon metabolism in cancer	5	0.001259	AKT2, KIT, AKT1, EGFR, FGFR1

hsa05214:Glioma	5	0.001263	AKT2, AKT1, IGF1, EGFR, IGF1R
hsa04917:Prolactin signaling pathway	5	0.001683	SRC, AKT2, AKT1, ESR1, ESR2
hsa05222:Small cell lung cancer	5	0.003178	RXRB, NOS2, AKT2, RARB, AKT1
hsa04914:Progesterone-mediated oocyte maturation	5	0.003305	HSP90AA1, AKT2, AKT1, IGF1, IGF1R
hsa05202:Transcriptional misregulation in cancer	6	0.004643	RXRB, MMP3, RARA, IGF1, MMP9, IGF1R
hsa04668:TNF signaling pathway	5	0.006539	CASP7, AKT2, MMP3, AKT1, MMP9
hsa05213:Endometrial cancer	4	0.00742	PDPK1, AKT2, AKT1, EGFR
hsa04150:mTOR signaling pathway	4	0.009642	PDPK1, AKT2, AKT1, IGF1
hsa04152:AMPK signaling pathway	5	0.009642	PDPK1, AKT2, AKT1, IGF1, IGF1R
hsa04550:Signaling pathways regulating pluripotency of stem cells	5	0.014806	AKT2, AKT1, IGF1, FGFR1, IGF1R
hsa04520:Adherens junction	4	0.01546	SRC, EGFR, FGFR1, IGF1R
hsa05152:Tuberculosis	5	0.031383	NOS2, SRC, VDR, AKT2, AKT1
hsa05231:Choline metabolism in cancer	4	0.038169	PDPK1, AKT2, AKT1, EGFR
hsa04931:Insulin resistance	4	0.044236	PDPK1, NOS3, AKT2, AKT1
hsa05145:Toxoplasmosis	4	0.045009	NOS2, PDPK1, AKT2, AKT1
hsa05219:Bladder cancer	3	0.046387	SRC, MMP9, EGFR

Supplementary Table S5. In silico analysis of toxicity of Tamarixetin using pkCSM (http://biosig.unimelb.edu.au/pkcsm/prediction_single/toxicity_1636099466.78)

**Model Name**	**Predicted Value**	**Unit**
AMES toxicity	No	Categorical (Yes/No)
Max. tolerated dose (human)	0.577	Numeric (log mg/kg/day)
hERG I inhibitor	No	Categorical (Yes/No)
hERG II inhibitor	No	Categorical (Yes/No)
Oral Rat Acute Toxicity (LD50)	2.407	Numeric (mol/kg)
Oral Rat Chronic Toxicity (LOAEL)	2.476	Numeric (log mg/kg_bw/day)
Hepatotoxicity	No	Categorical (Yes/No)
Skin Sensitisation	No	Categorical (Yes/No)
T.Pyriformis toxicity	0.299	Numeric (log ug/L)
Minnow toxicity	2.289	Numeric (log mM)

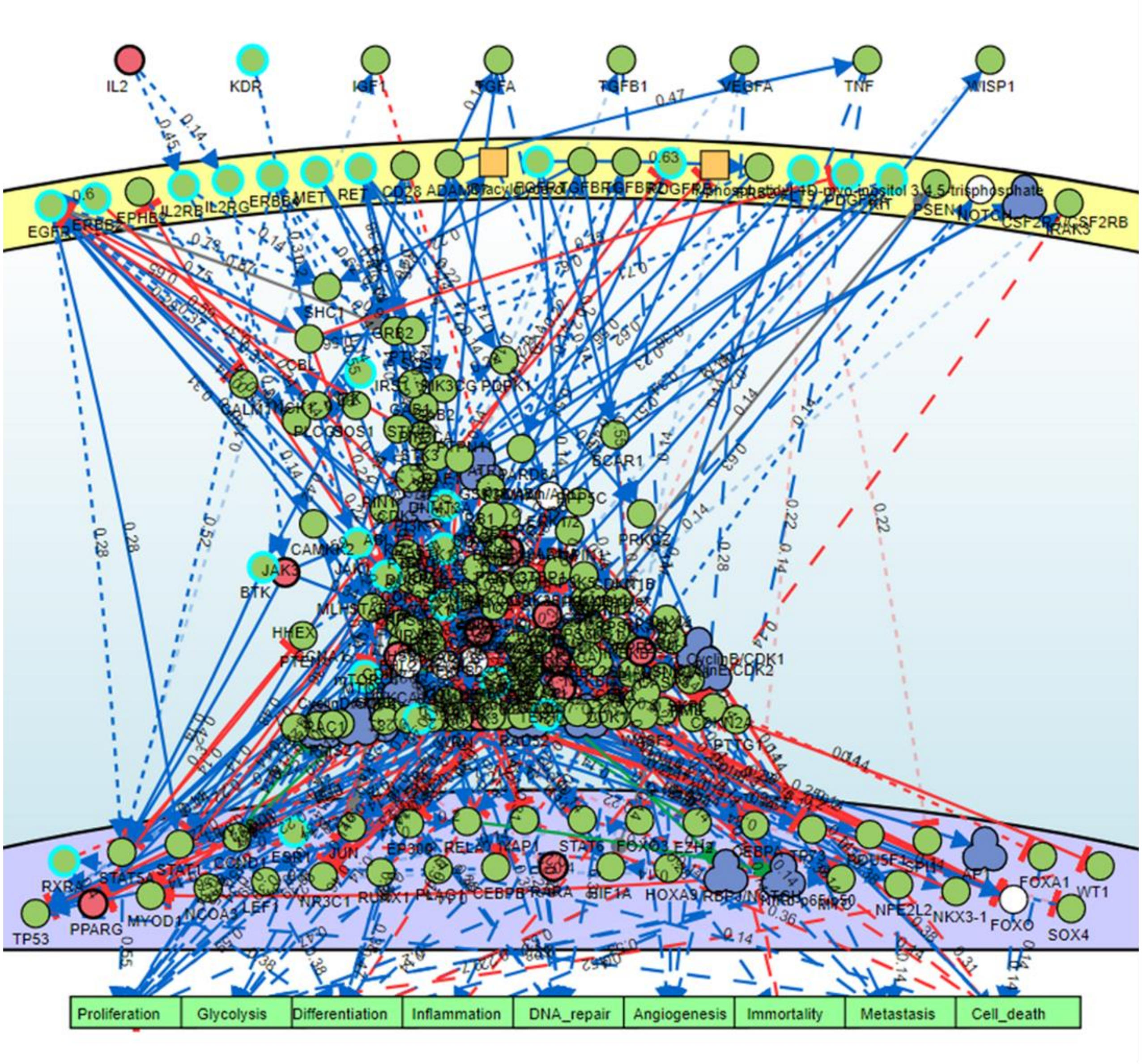

Supplementary Fig. S1. CancerGeneNet analysis for the identification of Tamarixetin target genes-cancer hallmarks associations.
